# Liquid chromatography mass spectrometry-based profiling of phosphatidylcholine and phosphatidylethanolamine in the plasma and liver of acetaminophen-induced liver injured mice

**DOI:** 10.1186/s12944-017-0540-4

**Published:** 2017-08-14

**Authors:** Ya-Nan Ming, Jing-Yi Zhang, Xiao-Lin Wang, Chun-Min Li, Si-Cong Ma, Zheng-Yang Wang, Xiao-Lin Liu, Xiao-Bo Li, Yi-Min Mao

**Affiliations:** 1 Division of Gastroenterology and Hepatology, Renji Hospital, School of Medicine, Shanghai Jiao Tong University, Shanghai Institute of Digestive Disease, Shanghai, China; 2 Department of Pharmacology, School of Medicine, Shanghai Jiao Tong University, Institute of Medical Sciences, Shanghai, China; 3 Department of Interventional Oncology, Renji Hospital, School of Medicine, Shanghai Jiao Tong University, Shanghai, China; 4 Department of Physiology and Pathophysiology, School of Basic Medical Sciences, Fudan University, Shanghai, 200032 China; 5 Division of Gastroenterology and Hepatology, Xinhua Hospital, School of Medicine, Shanghai Jiao Tong University, Shanghai, China

**Keywords:** Acetaminophen, Phosphatidylcholine, Phosphatidylethanolamine, Lipidomics, Liver injury

## Abstract

**Background:**

Acetaminophen (APAP) overdose is one of the most common causes of acute liver failure in many countries. The aim of the study was to describe the profiling of phosphatidylcholine (PC) and phosphatidylethanolamine (PE) in the plasma and liver of Acetaminophen -induced liver injured mice.

**Methods:**

A time course study was carried out using C57BL/6 mice after intraperitoneal administration of 300 mg/kg Acetaminophen 1 h, 3 h, 6 h, 12 h and 24 h. A high-throughput liquid chromatography mass spectrometry (LC-MS) lipidomic method was utilized to detect phosphatidylcholine and phosphatidylethanolamine species in the plasma and liver. The expressions of phosphatidylcholine and phosphatidylethanolamine metabolism related genes in liver were detected by quantitative Reverse transcription polymerase chain reaction (qRT-PCR) and Western-blot.

**Results:**

Following Acetaminophen treatment, the content of many PC and PE species in plasma increased from 1 h time point, peaked at 3 h or 6 h, and tended to return to baseline at 24 h time point. The relative contents of almost all PC species in liver decreased from 1 h, appeared to be lowest at 6 h, and then return to normality at 24 h, which might be partly explained by the suppression of phospholipases mRNA expressions and the induction of choline kinase (Chka) expression. Inconsistent with PC profile, the relative contents of many PE species in liver increased upon Acetaminophen treatment, which might be caused by the down-regulation of phosphatidylethanolamine N-methyltransferase (Pemt).

**Conclusions:**

Acetaminophen overdose induced dramatic change of many PC and PE species in plasma and liver, which might be caused by damaging hepatocytes and interfering the phospholipid metabolism in Acetaminophen -injured liver.

**Electronic supplementary material:**

The online version of this article (doi:10.1186/s12944-017-0540-4) contains supplementary material, which is available to authorized users.

## Background

As a major site for drug metabolism and elimination, the liver is susceptible to drug toxicity. Drug-induced liver injury (DILI) is a significant clinical problem and a challenge for drug development worldwide. Acetaminophen (N-acetyl-p-aminophenol, APAP) is commonly used as an over-the-counter analgesic and antipyretic drug known to be safe at therapeutic doses. However, APAP overdose has become one of the most common causes of acute liver failure in many countries [1]. APAP-induced liver injury is the most frequent drug hepatotoxicity and the most used experimental model of DILI. The mechanism of APAP-induced liver injury is complicated and not fully understood. The accumulation of N-acetyl-p-benzoquinone imine (NAPQI), the reactive and toxic metabolite of APAP, is considered as the main cause of liver injury induced by overdose of APAP. In mouse APAP-induced models and in human, the reaction of NAPQI with protein sulfhydryl groups of cysteine might trigger mitochondrial damage, oxidative stress, c-jun N-terminal kinase (JNK) activation, the nuclear DNA fragmentation and cell death [2–4].

In mammalian cells, phosphatidylcholine (PC) and phosphatidylethanolamine (PE) are the first and second most abundant phospholipid respectively. In liver, PC is the principal component of cellular membrane, a precursor of signaling molecules, and a key element of lipoproteins and bile. In addition to its structural role in membrane and as a substrate for methylation to PC in the liver, PE is also a substrate for anandamide synthesis, regulates membrane fusion, and supplies ethanolamine for glycosylphosphatidylinositol anchors of cell-surface signaling proteins. Previous studies over 50 years ago reported the contents of phosphatidylcholine (PC) and phosphatidylethanolamine (PE) were decreased,but the PE:PC ratio was elevated in the liver of rats challenged with CCl_4_ [5, 6]. Lipidomics is a powerful technology defined as the complete quantitative and molecular determination of lipid molecules isolated from cells, tissue or biological fluid [7–9]. During APAP-induced liver injury, lipidome is the overall results of cellular/subcellular dysfunction and alterations of larges molecules like proteins/enzymes/DNA, and consequently more easily correlated with the phenotype. The analysis of lipidome by lipidomics direct to a more detailed understanding of biochemical changes during DILI.

Recent years, emerging lipidomic techniques were used to describe comprehensive and global PC/PE profiles in plasma/liver of experimental animals with drug/chemical-induced liver injury. Several studies reported that serum levels of certain PCs/PEs were significantly changed in acute rat liver injury induced by chemicals such as APAP, CCL4, galactosamine and ricinine [10–12]. Cheng J et al. analyzed APAP-treated mice serum through LC-MS-based metabolomics, and they found that C20:4-LPC gradually declined with rising liver toxicity [13]. Xie T et al. observed the remodeling of PC/PE in liver of rats with Tripterygium wilfordii induced liver injury. The alternations comprised fatty acid composition of PC changed and increased Lyso PC and certain PE decreased, which might affect membrane fluidity, the inflammatory reaction, and mitochondria dysfunction respectively [14]. However, the alternation of individual PC/PE specie in both plasma and liver of APAP-injured mice and correlations of PCs/PEs between plasma and liver remains unknown. We already developed an LC-MS based lipidomic method for simultaneous detection of diverse lipids [15]. In the present study, using this high-throughput method, we compared the relative concentrations of phosphatidylcholine and phosphatidylethanolamine in the plasma and liver of the APAP-induced liver injured mice and saline-treated control mice at series of time-points, and then measured the expressions of genes involved in PC/PE metabolism in liver. The experimental work is focusing on the search for possible mechanisms leading to hepatotoxicity than on biomarkers indicating an APAP intoxication.

## Results

### Biochemical assays and H&E staining

**Fig. 1:**
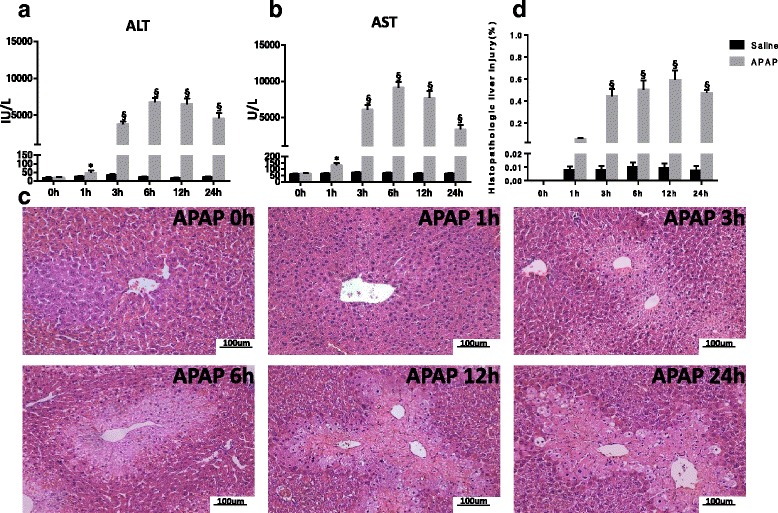
The activities of plasma ALT/AST and histopathology of the liver upon APAP administration. Mice were treated with saline or 300 mg/kg APAP for 0 h, 1 h, 3 h, 6 h, 12 h or 24 h (*n* = 6 ~ 8 for each time point). **a** and **b** Plasma levels of ALT and AST were measured by an automatic biochemical analyzer. **c** Liver sections from saline- or APAP-treated mice were stained with hematoxylin and eosin (H&E). **d** Statistic analysis of H&E staining image.*, *p* < 0.05; §, *p* < 0.001

Plasma levels of ALT and AST were measured to evaluate APAP-induced acute liver injury. As shown in Fig.1a and b, plasma ALT and AST levels increased in 300 mg/kg APAP induced acute liver injury mice model at all five time points (1 h, 3 h, 6 h, 12 h and 24 h). The most significant increase of ALT/AST was at 6 h time point. In histological evaluation, as shown in Fig.1c, APAP-treated mice displayed centrilobular hepatic necrosis, hyperemia of the hepatic sinus, presence of inflammatory infiltrate, pyknotic nucleus, cytoplasm vacuolization and loss of cell boundaries whereas saline-treated mice at any time point showed normal liver histology. The prominent morphological damage started at 3 h, deteriorated with time, and then attenuated at 24 h time point (Fig.1d).

### Phosphatidylcholine and phosphatidylethanolamine profiling in mice plasma analyzed by LC-MS

**Fig. 2:**
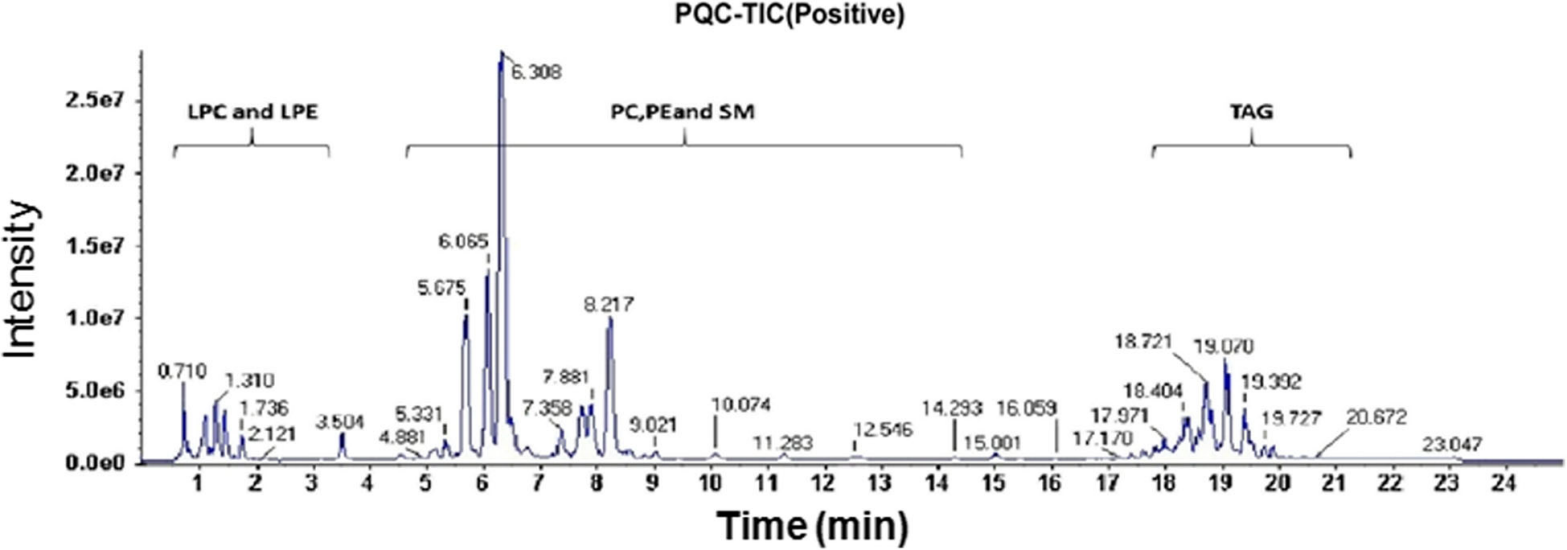
Total ions chromatogram (TIC) of mouse plasma sample

**Fig. 3:**
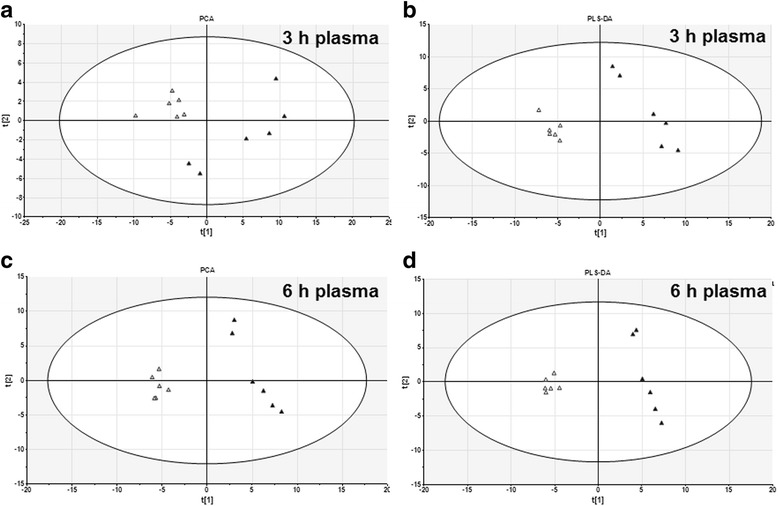
PCA and PLS-DA score plot based on the plasma PC and PE profiling of APAP-induced liver injured mice. Seventy-five variables (57 PC/LPC and 18 PE/LPE) at 3 h (**a**, **c**) and 6 h (**b**, **d**) time points were loaded into a PCA (**a**, **b**) model and a PLS-DA (**c**, **d**) model. The score plot indicated a clear separation between the saline-(△) and APAP-treated(▲) groups

The relative concentrations of 57 PC/LPC species and 18 PE/LPE species in plasma were simultaneously determined using LC-MS method. The typical positive total ion chromatograms (TIC) of mouse liver were shown in Fig. 2. The original data of PC/PE concentrations in plasma were shown in Supplemental Additional file 1: Table S1. The 75 phospholipids were loaded into a PCA model. As shown in Fig. 3a and c, the APAP-treated group could be clearly distinguished from the saline-treated group at 3 h time point as well as at 6 h time point according to the PCA score plots. These variables were further loaded into a PLS-DA model. As shown in Fig. 3b and d, the differences between saline- and APAP-treated samples were also depicted by PLS-DA score plot at 3 h time point or 6 h time point. The above score plots suggested that the metabolic pattern of PC/PE species in plasma was altered by APAP treatment at both 3 h and 6 h.

The ratio of lipid concentrations in APAP-treated group/lipid concentrations in saline-treated group at each time-point was calculated and represented as the relative concentrations of APAP-treated group in Additional file 1: Table S1. The fold-changes of PC/PE species in mouse plasma upon APAP treatment were also illustrated by heat map in Supplemental Additional file 2: Figure S1A (PC) and Figure S1B (PE).

**Fig. 4:**
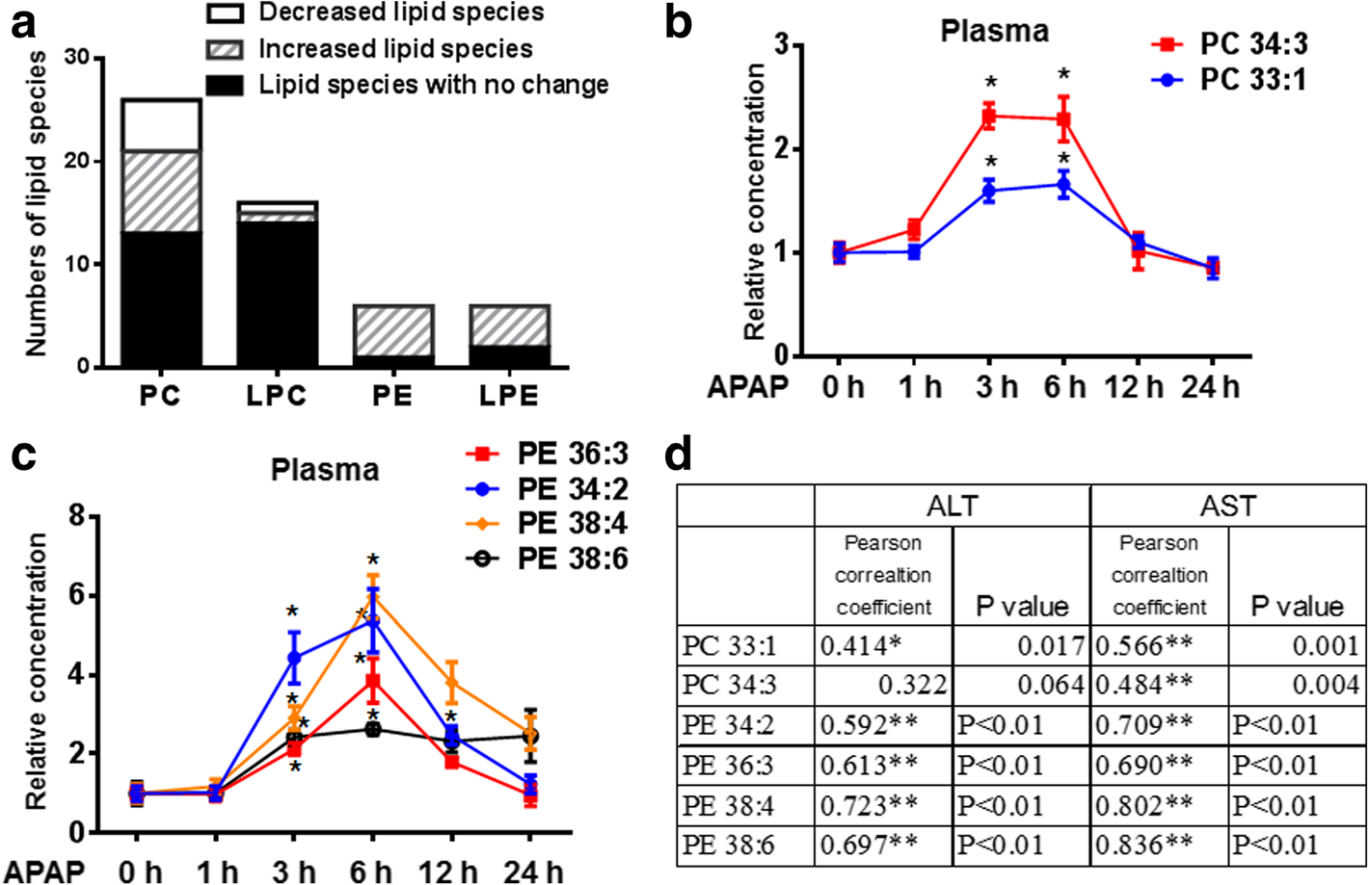
PC and PE profiles in the plasma of APAP-treated mice. **a** The numbers of decreased and increased PCs/LPCs/PEs/LPEs in plasma upon APAP-treatment were summarized. We defined lipid species with statistically significant decrease or increase at least at one time-point among 1 h, 3 h and 6 h as the decreased or increased lipid species respectively. **b** and **c** 2 PCs (PC 33:1 and PC 34:3) and 4 PEs (PE 34:2, PE 36:3, PE 38:4 and PE 38:6) were increased significantly in APAP-treated group compared with saline-treated group at both 3 h and 6 h time points. The relative concentrations of these 6 phospholipids in plasma of APAP induced liver-injured mice at 0 h, 1 h, 3 h, 6 h, 12 h and 24 h time points were presented. **d** Pearson’s correlation was performed to analyze the correlation between these 6 phospholipid species and liver enzymes (ALT/AST). *statistically significant compared with saline-treated group

Figure 4a summarized the numbers of decreased and increased PCs/LPCs/PEs/LPEs in plasma upon APAP-treatment. We defined lipid species with statistically significant increase or decrease at least at one time-point among 1 h, 3 h and 6 h as the increased or decreased lipid species respectively. The increased phospholipids are much more than decreased phospholipids in plasma upon APAP treatment. As shown in Fig. 4b and c, PC 33:1, PC 34:3, PE 34:2, PE 36:3, PE 38:4 and PE 38:6 were elevated significantly both at 3 h and 6 h time points upon APAP treatment. Pearson’s correlation was performed to analyze the correlation between these 6 phospholipid species and liver enzymes (ALT/AST) which were normally used to be elevated in DILI. As showed in Fig. 4d, the increases of these 6 phospholipids are positively correlated with the changes of ALT/AST.

### The profiles of PC and PE in mice livers analyzed by LC-MS

**Fig. 5:**
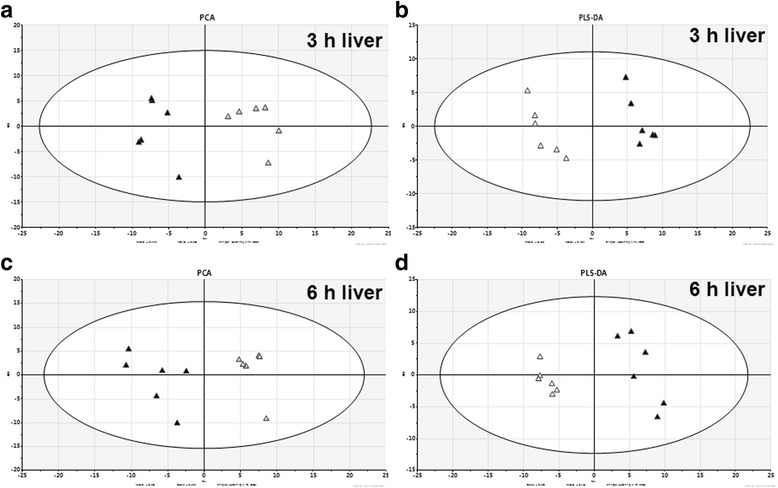
PCA and PLS-DA score plot based on the liver PC and PE profiling of APAP-induced liver injured mice. One hundred six variables (63 PC/LPC and 43 PE/LPE) at 3 h (**a**, **c**) and 6 h (**b**, **d**) time points were loaded into a PCA (**a**, **b**) model and a PLS-DA (**c**, **d**) model. The score plot indicated a clear separation between the saline-(△) and APAP-treated(▲) groups

To investigate the mechanism of PC/PE changing in plasma, we detected the PC/PE profiles in livers of APAP- and saline-treated mice by LC-MS. The levels of 63 PC/LPC species and 43 PE/LPE species in liver were simultaneously determined using LC-MS method. The analytical data’s of 106 PC/PE species were loaded into a PCA and a PLS-DA model. As shown in Fig. 5a/c (PCA model) and Fig. 5b/d (PLS-DA model), the differences between control group and APAP group were nearly identical in both data models. The above score plots suggested that the metabolic pattern of PC and PE in livers was altered by APAP treatment at both 3 h and 6 h.

**Fig. 6:**
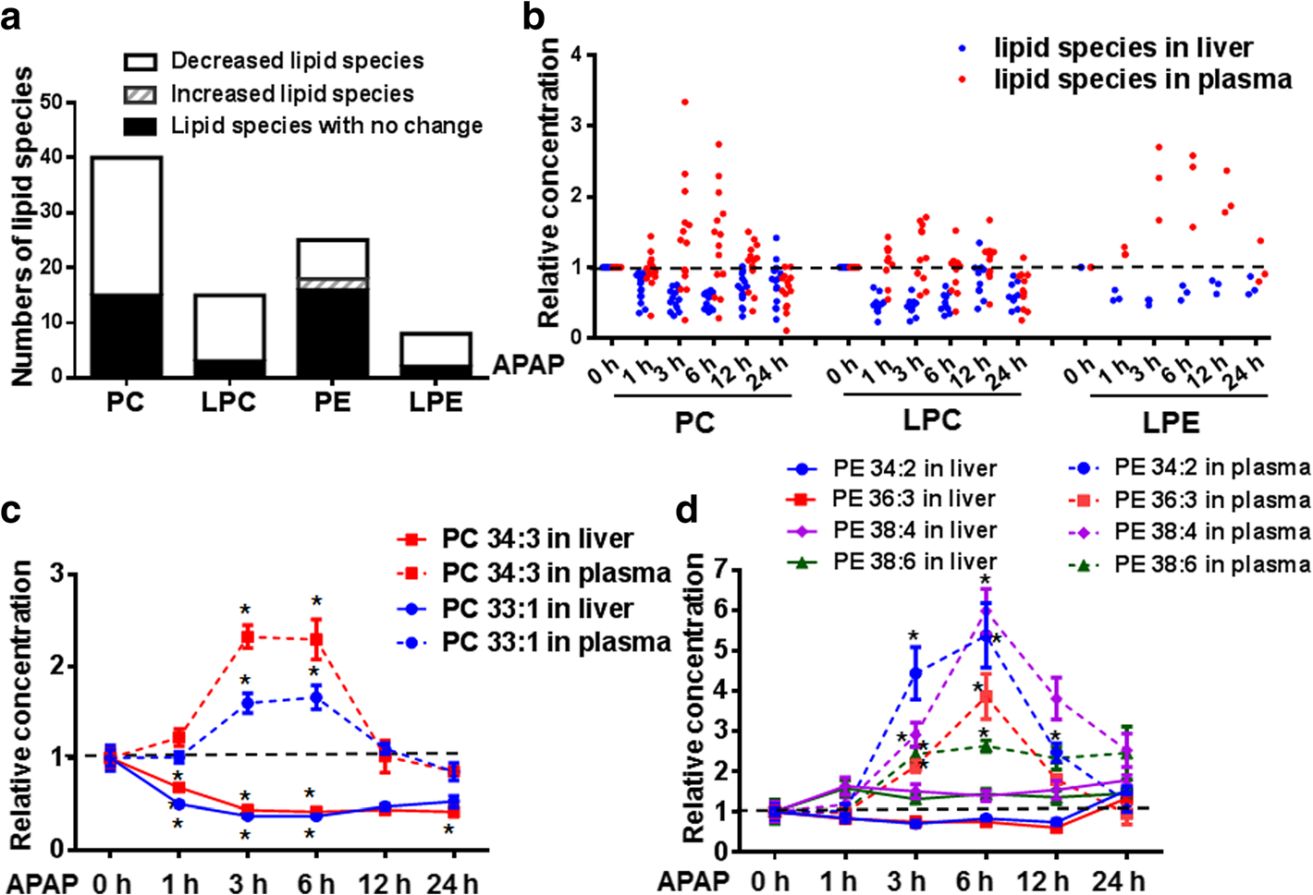
PC and PE profiles in the livers of APAP-treated mice. **a** The numbers of decreased and increased PCs/LPCs/PEs/LPEs in liver upon APAP-treatment were summarized. We defined lipid species with statistically significant decrease or increase at least at one time-point among 1 h, 3 h and 6 h as the decreased or increased lipid species respectively. **b** The decreased PCs/LPCs/PEs/LPEs in liver were picked out, and then Scatter Plot of these phospholipids both in liver and plasma were plotted. Because the significantly changed PEs in liver couldn’t be detected in plasma, PEs were not shown in Fig. 6b. **c** and **d** The relative concentrations of 2 PCs (PC 33:1 and PC 34:3) and 4 PEs (PE 34:2, PE 36:3, PE 38:4 and PE 38:6) in liver of APAP induced liver-injured mice at 0 h, 1 h, 3 h, 6 h, 12 h and 24 h time points were presented. *statistically significant compared with saline-treated group

The fold-changes of PC/PE species in mouse liver upon APAP treatment were also illustrated by heat map in Additional file 3: Figure S2A (PC) and figure S2B (PE). Figure 6a summarized the numbers of decreased and increased PCs/LPCs/PEs/LPEs in liver upon APAP-treatment. We defined lipid species with statistically significant decrease or increase at least at one time-point among 1 h, 3 h and 6 h as the decreased or increased lipid species respectively. The decreased phospholipids are much more than increased phospholipids in liver upon APAP treatment, which are opposite to profiles in plasma. We picked out the decreased PCs/LPCs/PEs/LPEs in liver, and then performed Scatter Plot of these phospholipids both in liver and plasma. As shown in Fig. 6b, most of decreased PCs and LPEs in liver might cause their increases in plasma. Because the significantly changed PEs in liver couldn’t be detected in plasma, PEs were not shown in Fig. 6b. Furthermore, the relative concentrations of the 6 increased plasma phospholipids in liver were plotted in Fig. 6c and d. PC 33:1 and PC 34:3 in liver decreased significantly. PE 34:2 and PE 36:3 in liver slightly decreased, while PE 38:4 and PE 38:6 in liver slightly increased.

### The expression pattern of PC and PE metabolism related genes in APAP-injured mice livers

**Fig. 7:**
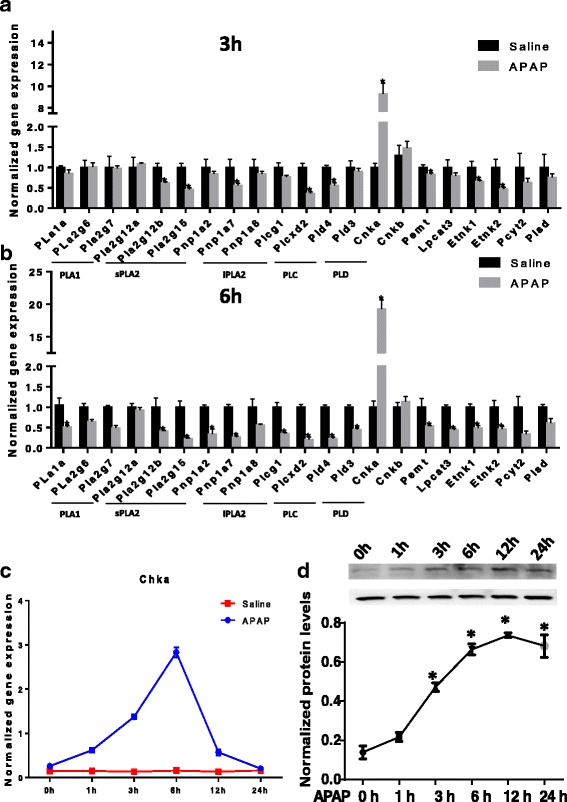
The expression pattern of PC and PE metabolism related genes in APAP-injured mice liver (**a**). qRT-PCR analysis of the mRNA expressions of 13 phospholipases in livers treated by APAP for 3 h and 6 h. **b** qRT-PCR analysis of the mRNA levels of PC/PE synthesis related genes in livers treated by APAP for 3 h and 6 h. **c** qRT-PCR analysis of the mRNA level of Chka in livers treated by APAP for 0 h, 1 h, 3 h, 6 h, 12 h or 24 h. **d** and **e** Western blot analysis of the protein levels of Chka in pooled liver samples treated by APAP for 0 h, 1 h, 3 h, 6 h, 12 h or 24 h. *n* = 6 per time point. *P < 0.05

To further investigate the mechanism of PC/PE changing in liver, we measured the expression of genes involved in PC/PE metabolism in liver tissue. There are over 40 different phospholipases in liver. Based on our previous RNAseq data, 13 phospholipases including 1 PLA1 (Pla1a), 8 PLA2 (Pla2g6, Pla2g7, Pla2g12a, Pla2g12b, Pla2g15, Pnpla2, Pnpla7, Pnpla8), 2 PLC (Plcg1, Plcxd2) and 2 PLD (Pld3, Pld4) are relatively abundant in mouse liver. The mRNA levels of these 13 phospholipases in livers of saline- or APAP-treated mice were detected by qRT-PCR. As shown in Fig. 7a, the concentration of mRNA of most phospholipases in liver decreased upon APAP treatment for 3 h or 6 h. We also measured the mRNA levels of PC/PE synthesis related genes. Among these genes, Pemt was decreased, while Chka was uniquely increased in APAP-injured livers (Fig. 7b). As shown in Fig. 7c, the induction of Chka was ~3.5-fold at 1 h, peaked at 6 h (~ 16-fold) and then went back to baseline at 24 h in APAP-injured livers. Western blot assays of the liver homogenates demonstrated Chka in livers upon APAP treatement was increased time-dependently, appeared to be greatest at the 12 h time point, which is about several hours-delayed compared with Chka mRNA induction (Fig. 7d).

## Discussion

In the present study, we utilized the high-throughput LC-MS lipidomic method to acquire PC and PE profiles both in plasma and liver of APAP-induced liver injured mice at different time points after dosing.

The mice model of APAP-induced liver injury mostly resembles the human pathophysiology of both liver injury and recovery [16]. In our study, the sub-lethal dose of APAP (300 mg/kg) was used to induce liver injury in mice. In this model, the most significant increase of ALT/AST is at 6 h time point. At 24 h time point, the liver function presented the recovery situation verified by enzymatic and morphological evaluation. Consistently, the most dramatic change of PC/PE profiles occurred at 3 h and 6 h time points, so varied PC/PE at both 3 h and 6 h time points were emphasized in our analysis.

As lipids serve many important functions, take part in several biochemical reactions and integrate diverse metabolic pathways, any alteration in lipids will reflect and affect cellular functions. Based on our results, the content of many PC/PE species in plasma increased from 1 h time point, peaked at 3 h or 6 h, and tended to return normality at 24 h time point. PC 33:1, PC 34:3, PE 34:2, PE 36:3, PE 38:4 and PE 38:6 were elevated significantly both at 3 h and 6 h time points upon APAP treatment. The increases of these 6 phospholipids were positively correlated with the changes of ALT/AST. The alternation of PC/PE lipidome in plasma might be caused by damaging hepatocytes and/or interfering the lipid metabolism in liver. Based on our results, following an intoxication with APAP, many PC/PE species such as PC 33:1, PC 34:3, PE 34:2 and PE 36:3 in liver tissue decreased from 1 h, appeared to be lowest at 6 h, and had the tendency back to normal status at 24 h time point, which was opposite to their profile in plasma. Therefore, the increase of these phospholipids in plasma might be released by damaged hepatocytes in APAP-injured livers. However, some PEs increased in APAP-injured livers, which might cause the induction of certain PEs such as PE 38:4 and PE 38:6 in plasma.

The contents of PC/PE in liver rely on their degradation and synthesis. The degradation of PC/PE results from the action of phospholipases. There are various phospholipases (PLA1, PLA2, PLC and PLD) that exhibit substrate specificities for different positions in phospholipids. Phospholipase A2 (PLA2) hydrolyzes the sn-2 ester bond in PC/PE, forming arachidonic acid and lyso-PC/lyso-PE [17–19]. These bioactive lipid mediators play important roles in inflammation, phospholipid metabolism, and signal transduction, which participate in the progression of DILI. The PLA2 superfamily includes over twenty groups comprising such main types as the secreted sPLA2, cytosolic cPLA2, calcium-independent iPLA2, and so on. PC and PE also could be degraded by PLA1, PLC and PLD [20]. The decrease of PC contents might be the consequence of increased activities of phospholipases. Previous studies demonstrate that overdose of APAP may activate cPLA_2_ and sPLA_2_ [21–23], which are involved in APAP toxicity. The activated phospholipases degraded PC in livers. Our present study indicated that the mRNA levels of phospholipases were suppressed by APAP treatment, which might be the adaptive protective reaction for the remaining alive hepatocytes.

Except for degradation, the contents of phospholipids in liver are also affected by their synthesis. The major pathway of PC synthesis is the CDP-choline pathway, also referred to as the “Kennedy pathway”, which requires choline and three enzymes including choline kinase (Chka and Chkb), CTP:phosphocholine cytidylyltransferase (Pcyt) and diacylglycerol cholinephosphotransferase (Chpt). In liver, PC can also be generated endogenously in a second pathway via PE methylations catalyzed by hepatic phosphatidylethanolamine N-methyltransferase (PEMT), which produces about one third of the PC in liver. PC also could be synthesized by reacylation of lyso-PC with lyso-PC acyltransferase (LPCAT) [24, 25]. We observed that the mRNA and protein levels of Chka, the rate-limiting enzyme of synthesizing PC de novo, were induced dramatically in APAP-injured mice. Chka was found to play a vital role in many biological signaling pathway, such as androgen receptor (AR) chaperone [26], and cell proliferation and carcinogenesis. Extensive studies of the structure and function of Chka show that the distal of the Chka promoter regions sequence is similar to the consensus of activated protein-1 binding site [27].In addition, APAP overdose causes nuclear accumulation of Hypoxia inducible factor-1 (HIF-1) in mouse livers as early as 1 h after treatment [28]. The expression of Chka was identified to be due to the regulation of transcriptional expression of HIF-1 [29]. HIF-1 deficiency mice were protected from APAP hepatotoxicity at 6 h, but severe liver injury was observed at 24 h, suggesting that HIF-1 is involved in the early stage of APAP toxicity [30]. Thus, the altered characteristic of Chka may be related to the alteration of associated transcriptional factors. The increased activities of phospholipases, decreased phospholipases mRNA expressions, and increased Chka mRNA/protein expression might partly explain why the contents of PC species in livers increased after APAP dosing and tended to recover at 24 h time point. PE is synthesized by four different pathways, the two quantitatively major of which are the CDP-ethanolamine pathway that PE catalyzed by ethanolamine kinase (ETNK1 and ETNK2) in the ER and the PS decarboxylation pathway that catalyzed by phosphatidylserine decarboxylase (PISD) in mitochondria [31]. The increase of some PE species in APAP-injured livers might be involved in the suppression of Pemt which is responsible one third of PC synthesis in liver. The dramatic decrease of Pemt may lead the accumulation of PE in APAP-injured liver.

Our study for the first time determined the dynamic PC/PE changes both in plasma and liver that occurred in APAP-induced liver injury by lipidomics. This time-course study provides a deeper understanding of the metabolic changes observed in APAP-induced liver injury. We took the plasma PC/PE lipidome, liver tissue PC/PE lipidome, and PC/PE metabolism-related gene expressions into consideration intergratedly. The comprehensive analysis might contribute to a better understanding the effect of APAP on lipid metabolism, hence finding potential therapeutic targets. In future studies, the concentration dependent changes of the PC/PE species in mice upon APAP treatment should be observed, which might provide valuable information for judging prognosis of DILI. Huo T et al. performed both UPLC-MS and (1)HNMR-based metabonomics analysis of serum samples from 34 epileptic patients after valproate sodium treatment, and found that certain LPCs such as LPC 16:0, LPC 18:0, LPC 18:1 and LPC 18:2 decreased in valproate sodium induced liver injury [32]. The phospholipid profile of serum in human intoxicated with APAP also might be further observed by lipidomic techniques.

## Conclusions

These results suggest that a novel targeted lipidomic method based on the metabolic profiling of phospholipid analyzed by LC-MS provides a better understanding the role of lipid metabolism in APAP-induced injured liver and serum, which might provide valuable information for judging prognosis of DILI and therapeutic targets.

## Methods

### Animals

C57BL/6 males aged 8–10 weeks and weighed 22–25 g at the time of the experimental procedures were purchased from the Shanghai Laboratory Animal Center, Chinese Academy Sciences (Shanghai, China). All mice were housed in microisolator cages under humidity- (50% ± 5%) and temperature- (24° ± 2 °C) controlled specific pathogen-free conditions with 12 h light/dark cycle. The mice were maintained on with free access to water and standard irradiated sterile chow.

### Treatment of mice

Fresh acetaminophen (Sigma Aldrich, USA) solution was prepared for each experiment by dissolving acetaminophen in saline warmed to 59 °C. Mice were fasted for 16 h and then injected intraperitoneally (i.p.) with saline or acetaminophen at 300 mg/kg (body weight) and food restored. APAP-treated mice were sacrificed and collected liver and plasma samples at the indicated time points (0 h, 1 h, 3 h, 6 h, 12 h, and 24 h, *n* = 6 ~ 8 each time point). Some mice were treated with saline as parallel controls at the same time points (1 h, 3 h, 6 h, 12 h, and 24 h, *n* = 6 ~ 8 each time point). Appropriate volume of PMSF stock solution in isopropanol (100 mM) were added to freshly collected mouse plasma (5 mM in plasma) to stabilize the plasma lipidome. The livers were frozen in −80 °C for later lipid/total RNA/protein extraction or fixed in 4% paraformaldehyde for tissue sections.

### Lipidomics analysis

The lipids of plasma (50 uL) and liver samples (200 mg) were extracted using liquid-liquid methyl tert-butyl ether (MTBE) extraction protocols developed by our group [15]. Briefly, endogenous free lipids including PE (17:0/17:0), PC (19:0/19:0), LPE 14:0, LPC 17:0 were selected as the internal standard for their corresponding lipid classes. The lipids of the mixture of the above mixed internal standard solution and samples were extracted twice using MTBE, dried and finally reconstituted in 200 μL in the mixture of isopropanol/acetonitrile/water (75:20:5, *v*/v/v), then subjected to LC-MS analysis. An HPLC-quadrupole-time of flight hybrid mass system, which consisted of a LC-30 AD UFLC system (Shimadzu, Kyoto, Japan) and a TripleTOF 5600þ quadrupole time of flight mass spectrometer (AB SCIEX, MA, USA), was used for lipidomic analysis. Chromatography was performed on a charged surface hybrid (CSH™) C18, 2.1*100 mm, 1.7 um UPLC column (Waters Corporation, USA) and subjected to a gradient elution as described previously. The eluents were monitored in positive electronic ion spray ESI mode. The lipid profiling was performed using the previous methods [33]. Briefly, the extracted ions chromatogram (EIC) of each lipid specie was created by applying the molecular weight (MW) lists (MW of quasi-molecular ions) generated from Lipidview™ software (AB SCIEX, MA, USA). The peaks found in EIC were screened and identified according to the rules, including exact mass accuracy (< 5 ppm), specific fragment (PC and LPC have the specific product of 184.1; PE and LPE have the specific neutral loss of 141), retention time and isotope distribution pattern (similarity to the theoretical pattern). After corresponding IS correction, the areas of the identified peaks were used for quantitation.

### Biochemical assays and histopathology

Alanine aminotransferase (ALT) and aspartate aminotransferase (AST) were measured by an automatic biochemical analyzer (SIEMENS ADVIA 1800: SIEMENS Healthcare Diagnostics, USA). Liver sections from saline- or APAP-treated mice were fixed in 4% paraformaldehyde overnight and embedded in paraffin wax, sliced at 5 mm thickness and stained with hematoxylin and eosin (H&E).

### Real-time RT-PCR (qRT-PCR) analysis

Total RNA was prepared from mouse livers using Trizol reagent (Life Technologies, Thermo Fisher Scientific). Reverse transcription (RT) was performed using RevertAid™ First Strand cDNA Synthesis Kit (Fermentas) according to the manufacturer’s instructions. Relative expressions of indicated genes were determined by SYBR Green-based real-time PCR using Actb as an internal standard. A relative standard curve was used to calculate expression levels. Primers used for gene expression studies are listed in Table 1, and referenced in the primer bank [34].

**Table 1:** Primers sets used for qPCR

Gene name	Genbank accession NM	Primer (5′-3′)
Actb	NM_007393.5	Forward: CGTGCGTGACATCAAAGAGAA Reverse: GCTCGTTGCCAATAGTGATGA
Pla1a	NM_134102.4	Forward: GGACTTTCTGTACTGCCCCT Reverse: CGCAGGCTATTTTCAGGTCC
Pla2g12a	NM_001286948.1	Forward: GAAGCCTGTTCCACGCTATG Reverse: CACTTGGTCAGGGAAGGGAT
Pnpla2	NM_001163689.1	Forward: ATGGTGCCCTATACTCTGCC Reverse: TCTTCAGGGACATCAGGCAG
Plcg1	NM_021280.3	Forward: CCAGCAGAGAAACATGGCTC Reverse: TCTTCCTCCTGAGCTGGTTG
Pld3	NM_001317355.1	Forward: TAAAGGTTCGCATCGCTGTG Reverse: CTGATCCACCACCCAGAACT
Pla2g12b	NM_023530.2	Forward: GGTGTCGATATGGAAAGGCG Reverse: AACACTTGGTCATTGCTGGG
Pnpla7	NM_146251.4	Forward: ATCAAACAACGCCTGGGTTC Reverse: GCCTGATACAGCACAATCCG
Plcxd2	NM_001134480.1	Forward: CTTCATCCACGGGCTCTTTG Reverse: TCTTGAGGGTGCTGAGTGAG
Pld4	NM_178911.4	Forward: CAAGTGCCCATGAAACAGCT Reverse: GCACCAAGTTCCTTCACCTG
Pla2g6	NM_001199023.1	Forward: GTCCTGCTGCTCTGTAATGC Reverse: TCCTTGCTGTGGATCTGGTT
Pnpla8	NM_026164.2	Forward: TGGTGGAGGAACAAGAGGTG Reverse: TATGGCCCCTGTGCTTACTC
Pla2g15	NM_133792.2	Forward: GGCCTCCTGTTACCTCTGTT Reverse: GGTTACCCAAATCACCAGGC
Pla2g7	NM_013737.5	Forward: CAGCTCAAGATCAAGGTCGC Reverse: CAGCTTGCAGGAGTTGTCAG
Pemt	NM_001290011.1	Forward: CGAGATGGGAGCAGAGAACT Reverse: ATCTTGGGCTGGCTCATCAT
Lpcat3	NM_145130.2	Forward: CCAGGGAAGATGCCAAACAG Reverse: TAGTCGTCTGTGATGTGGGG
Chka	NM_001271496.1	Forward: CTTCGCGAGGACCAGTTC Reverse: CTATGGAGTCTGGCAGGGAG
Pcyt2	NM_024229.2	Forward: TGGTGCGATGGCTGCTATG Reverse: CCCTTATGCTTGGCAATCTCC
Pisd	NM_177298.3	Forward: CATACTGCTCCTGTCCGATCC Reverse: TTCCGTTCCCTGTACTTCTCATA
Etnk1	NM_029250.2	Forward: TGAGGATTTACGGCAACAAGAC Reverse: CAGTCCGTTATTAAAGGTGCAGT
Etnk2	NM_175443.5	Forward: CGGTGGAACAGGACGACATC Reverse: AGGCCAATAGCTTGTTGGTGA
Chkb	NM_007692.6	Forward: GCCGAGCCTATCAGTGGTG Reverse: GTAGTGAGCATCGGAAGAGCA

### Western blot analysis

Total protein was extracted from treated livers using radioimmune precipitation assay (RIPA) lysis buffer. The Chka protein level was detected by primary antibody against Chka (Proteintech Group, Inc. Rosemont, IL, USA) using Western blot assay according to published methods [35]. β-actin was used as loading control.

### Data analysis

MultiQuant™ software (Version 2.1.1, AB SCIEX, MA, USA), calculated the peak area ratios of lipid species and their corresponding internal standards, as the concentrations of lipid species. PCA and PLS-DA were performed using SIMCA-P 12.0 software (Umetrics, Umea, Sweden). The original data of PC/PE concentrations and the fold changes of APAP-treated mice compared with saline-treated mice at indicated time points in plasma and liver were shown in Supplemental tables (Additional file 1: Table S1 and Additional file 4: Table S2) respectively. The original data were presented as the mean ± SD. To compare the concentrations of PC/PE between the APAP-treated group and saline-treated group in each indicated time point, statistical analysis was performed with t-tests for unpaired data, the statistical significance was corrected for multiple comparisons using the Holm-sidak method by using GraphPad Prism 6.0.

## Additional files

Additional file 1: Table S1. The original data of PC/PE concentrations and the fold changes of APAP-treated mice compared with saline-treated mice at indicated time points in plasma. The original data were presented as the mean ± SD. The lipid species with statistical significance were labeled with red. (DOCX 32 kb)

Additional file 2: Figure S1. Heat map for PC and PE profiles in the plasma of APAP-treated mice. The mean of the concentration of PC(A)/PE(B) in plasma of APAP-treated mice at indicated time point (*n* = 6~8 for each time point) were normalized to the saline-treated group at the same time point. The color of each section is proportional to the relative concentration of phospholipids as shown in the color bar (blue-black-yellow colors correspond to low-moderate-high levels respectively). Rows: phospholipid species; columns: samples at indicated time point. (TIFF 48 kb)

Additional file 3: Figure S2. Heat map for PC and PE profiles in the livers of APAP-treated mice. The mean of the concentration of PC(A)/PE(B) in livers of APAP-treated mice at indicated time point (*n* = 6~8 for each time point) were normalized to the saline-treated group at the same time point. The color of each section is proportional to the relative concentration of phospholipids as shown in the color bar (blue-black-yellow colors correspond to low-moderate-high levels respectively). Rows: phospholipid species; columns: samples at indicated time point. (TIFF 57 kb)

Additional file 4: Table S2. The original data of PC/PE concentrations and the fold changes of APAP-treated mice compared with saline-treated mice at indicated time points in liver. The original data were presented as the mean ± SD. The lipid species with statistical significance were labeled with red. (DOCX 39 kb)
