# Supplementary material for: Liquid chromatography mass spectrometry-based profiling of phosphatidylcholine and phosphatidylethanolamine in the plasma and liver of acetaminophen-induced liver injured mice
Source: Lipids Health Dis. 2017 Aug 14;16:153. doi: 10.1186/s12944-017-0540-4 (PMC5556666; doi:10.1186/s12944-017-0540-4)
Supplement: Supplementary file 1 — The original data of PC/PE concentrations and the fold changes of APAP-treated mice compared with saline-treated mice at indicated time points in plasma. The original data were presented as the mean ± SD. The lipid species with statistical significance were labeled with red. (DOCX 32 kb) [file 12944_2017_540_MOESM1_ESM.docx]

|  | N/A | Saline | | | | | APAP (300 mg/kg) | | | | | APAP/Saline | | | | | |
| --- | --- | --- | --- | --- | --- | --- | --- | --- | --- | --- | --- | --- | --- | --- | --- | --- | --- |
| Plasma PC/PE | 0 h | 1 h | 3 h | 6 h | 12 h | 24 h | 1 h | 3 h | 6 h | 12 h | 24 h | 0 h | 1 h | 3 h | 6 h | 12 h | 24 h |
| PC 30:0 | 0.00916±0.00119 | 0.00864±0.00082 | 0.01027±0.00077 | 0.01021±0.00087 | 0.00994±0.00102 | 0.0076±0.00076 | 0.01021±0.00143 | 0.01388±0.00297 | 0.01382±0.00269 | 0.01708±0.00361 | 0.00953±0.00085 | 1.000 | 1.182 | 1.352 | 1.353 | 1.718 | 1.254 |
| PC 32:0 | 0.80832±0.11396 | 0.77952±0.04633 | 0.84417±0.0645 | 0.84795±0.08115 | 0.86386±0.08571 | 0.82633±0.02575 | 0.73398±0.08161 | 0.81798±0.1592 | 0.77193±0.10813 | 1.13961±0.23201 | 0.83387±0.17708 | 1.000 | 0.942 | 0.969 | 0.910 | 1.319 | 1.009 |
| PC 32:1 | 0.28019±0.05061 | 0.23013±0.03284 | 0.28718±0.04263 | 0.27403±0.03931 | 0.23314±0.07442 | 0.15476±0.00194 | 0.18108±0.04394 | 0.59662±0.0682 | 0.56516±0.18952 | 0.2303±0.08043 | 0.15593±0.02304 | 1.000 | 0.787 | 2.078 | 2.062 | 0.988 | 1.008 |
| PC 32:2 | 0.07534±0.01805 | 0.06683±0.01477 | 0.0827±0.01217 | 0.08476±0.01083 | 0.07763±0.03064 | 0.06111±0.01299 | 0.09641±0.03257 | 0.27657±0.0341 | 0.23274±0.08525 | 0.11693±0.07003 | 0.04126±0.00663 | 1.000 | 1.443 | 3.344 | 2.746 | 1.506 | 0.675 |
| PC 33:1 | 0.02792±0.00293 | 0.02494±0.00262 | 0.03116±0.00509 | 0.02741±0.00261 | 0.02358±0.00386 | 0.01932±0.00106 | 0.02515±0.00364 | 0.04989±0.00818 | 0.04561±0.00935 | 0.026±0.00384 | 0.01644±0.00316 | 1.000 | 1.008 | 1.601 | 1.664 | 1.102 | 0.851 |
| PC 33:2 | 0.20259±0.036 | 0.187±0.04556 | 0.20691±0.0505 | 0.17158±0.03068 | 0.16265±0.0357 | 0.25204±0.04012 | 0.20798±0.04802 | 0.33798±0.0473 | 0.3024±0.05024 | 0.22718±0.05928 | 0.15839±0.05558 | 1.000 | 1.112 | 1.634 | 1.762 | 1.397 | 0.628 |
| PC 34:0 | 0.1541±0.01374 | 0.16386±0.03552 | 0.16365±0.01165 | 0.17627±0.04388 | 0.13715±0.00628 | 0.22711±0.01611 | 0.12249±0.02237 | 0.13295±0.03234 | 0.10221±0.02618 | 0.19021±0.04506 | 0.12799±0.06291 | 1.000 | 0.748 | 0.812 | 0.580 | 1.387 | 0.564 |
| PC 34:1 | 12.04101±1.94269 | 11.14337±0.90646 | 14.08158±2.18877 | 13.77517±1.51964 | 11.33188±1.67248 | 9.34187±0.66055 | 9.45083±0.72794 | 21.3±7.21043 | 20.23±4.56667 | 12.82625±1.69228 | 8.229±0.96762 | 1.000 | 0.848 | 1.513 | 1.468 | 1.132 | 0.881 |
| PC 34:2 | 110.9±13.71202 | 112.28987±7.45643 | 112.8907±9.16367 | 120.10145±7.08553 | 111.40672±8.49853 | 117.65631±7.99916 | 103.85833±8.15331 | 140.56667±14.27118 | 142.68571±19.28846 | 114.735±8.72124 | 80.75333±14.64145 | 1.000 | 0.925 | 1.245 | 1.188 | 1.030 | 0.686 |
| PC 34:3 | 1.10567±0.22774 | 0.97781±0.15231 | 1.17131±0.29682 | 1.0073±0.13707 | 0.8566±0.28168 | 0.66967±0.10768 | 1.1961±0.22314 | 2.7255±0.34977 | 2.31214±0.57663 | 0.87188±0.42824 | 0.5736±0.02606 | 1.000 | 1.224 | 2.326 | 2.296 | 1.018 | 0.857 |
| PC 35:1 | 0.02911±0.00252 | 0.02687±0.00264 | 0.03192±0.00467 | 0.03042±0.00204 | 0.02813±0.00584 | 0.03128±0.0048 | 0.02862±0.00364 | 0.043±0.01111 | 0.03979±0.00833 | 0.03509±0.00332 | 0.02499±0.00474 | 1.000 | 1.065 | 1.347 | 1.309 | 1.247 | 0.799 |
| PC 35:2 | 0.71377±0.24321 | 0.62912±0.21827 | 0.85674±0.27797 | 0.67259±0.19657 | 0.71951±0.0708 | 0.72421±0.04097 | 0.66585±0.05953 | 0.81442±0.06256 | 0.7908±0.12994 | 0.69205±0.07665 | 0.5369±0.15107 | 1.000 | 1.058 | 0.951 | 1.176 | 0.962 | 0.741 |
| PC 36:0 | 0.00686±0.00089 | 0.00699±0.00041 | 0.00711±0.00048 | 0.00694±0.00072 | 0.00597±0.00029 | 0.00908±0.00068 | 0.0061±0.00136 | 0.00578±0.00074 | 0.00472±0.00092 | 0.0069±0.00089 | 0.00474±0.00193 | 1.000 | 0.873 | 0.813 | 0.680 | 1.156 | 0.522 |
| PC 36:1 | 1.70871±0.14424 | 1.58159±0.05432 | 1.69191±0.06892 | 1.61293±0.08695 | 1.43278±0.13483 | 1.88278±0.12926 | 1.38183±0.15126 | 1.49917±0.20822 | 1.457±0.36886 | 1.65775±0.18475 | 1.26467±0.19404 | 1.000 | 0.874 | 0.886 | 0.904 | 1.157 | 0.672 |
| PC 36:2 | 39.4±3.5192 | 37.40198±2.09442 | 41.34929±3.56699 | 39.9726±4.22379 | 33.36208±5.2945 | 67.46976±8.51884 | 28.82±3.47153 | 36.30333±4.05736 | 38.15429±8.00791 | 39.9925±8.98939 | 28.16±9.55713 | 1.000 | 0.771 | 0.878 | 0.955 | 1.199 | 0.417 |
| PC 36:3 | 3.78254±0.79574 | 3.46423±0.43833 | 4.25126±0.54372 | 3.87098±0.46208 | 3.51678±0.67716 | 5.97137±0.99264 | 3.41183±0.48828 | 5.9025±0.5406 | 5.821±1.21047 | 3.87075±0.56174 | 2.59233±0.7338 | 1.000 | 0.985 | 1.388 | 1.504 | 1.101 | 0.434 |
| PC 36:4 | 28.72338±4.37853 | 25.69417±1.23833 | 31.58806±4.95575 | 35.58868±4.57361 | 30.08247±5.07883 | 18.70716±1.38576 | 36.37333±3.96234 | 52.56167±10.03512 | 47.78714±9.71286 | 36.2875±11.54879 | 14.99333±2.0512 | 1.000 | 1.416 | 1.664 | 1.343 | 1.206 | 0.801 |
| PC 36:5 | 0.59394±0.11108 | 0.54042±0.04911 | 0.74638±0.08464 | 0.9072±0.11067 | 0.87427±0.09071 | 0.5481±0.02698 | 0.36577±0.03548 | 0.33307±0.1124 | 0.34811±0.13267 | 0.7998±0.2037 | 0.3816±0.14802 | 1.000 | 0.677 | 0.446 | 0.384 | 0.915 | 0.696 |
| PC 37:2 | 0.35248±0.05913 | 0.35001±0.05825 | 0.31634±0.03995 | 0.32816±0.05779 | 0.29396±0.0206 | 0.6077±0.07538 | 0.32277±0.06247 | 0.22027±0.03992 | 0.18969±0.0252 | 0.19178±0.05335 | 0.21661±0.14591 | 1.000 | 0.922 | 0.696 | 0.578 | 0.652 | 0.356 |
| PC 37:4 | 0.12878±0.00297 | 0.09488±0.04131 | 0.12769±0.01279 | 0.14158±0.01829 | 0.12124±0.01007 | 0.07178±0.00503 | 0.24013±0.04091 | 0.24462±0.03626 | 0.2114±0.03534 | 0.17003±0.06944 | 0.07686±0.00853 | 1.000 | 2.532 | 1.916 | 1.493 | 1.402 | 1.071 |
| PC 37:6 | 0.01888±0.00329 | 0.01664±0.00215 | 0.02271±0.0036 | 0.02272±0.00296 | 0.02141±0.00514 | 0.01606±0.00109 | 0.0221±0.00309 | 0.03423±0.00834 | 0.02703±0.00849 | 0.02289±0.00713 | 0.01128±0.00351 | 1.000 | 1.328 | 1.508 | 1.190 | 1.069 | 0.702 |
| PC 38:2 | 0.16592±0.03601 | 0.14015±0.03944 | 0.15337±0.0276 | 0.14839±0.02685 | 0.13422±0.0208 | 0.61933±0.08878 | 0.04475±0.00743 | 0.03948±0.00918 | 0.0423±0.01064 | 0.05183±0.02593 | 0.06787±0.03415 | 1.000 | 0.319 | 0.257 | 0.285 | 0.386 | 0.110 |
| PC 38:3 | 0.9722±0.11913 | 0.90704±0.11618 | 0.90604±0.03929 | 0.91819±0.16914 | 0.79093±0.11138 | 2.12616±0.22392 | 0.72368±0.10296 | 0.62655±0.03479 | 0.6834±0.10841 | 0.78523±0.12081 | 0.62143±0.16682 | 1.000 | 0.798 | 0.692 | 0.744 | 0.993 | 0.292 |
| PC 38:4 | 9.402±1.03957 | 8.28794±0.34097 | 10.07115±0.71595 | 10.31562±1.53219 | 8.54897±0.79827 | 5.56273±0.31775 | 11.3335±1.71864 | 11.60833±1.14293 | 12.345±2.8432 | 10.95475±3.1343 | 5.55167±0.63561 | 1.000 | 1.367 | 1.153 | 1.197 | 1.281 | 0.998 |
| PC 38:5 | 0.96558±0.10018 | 0.92008±0.06397 | 1.13553±0.15048 | 1.1596±0.1584 | 0.99973±0.08998 | 0.60099±0.01939 | 1.00543±0.10459 | 1.15792±0.1579 | 1.1606±0.20613 | 0.96564±0.33716 | 0.41197±0.07235 | 1.000 | 1.093 | 1.020 | 1.001 | 0.966 | 0.686 |
| PC 38:6 | 32.0882±3.10481 | 31.08177±2.84796 | 38.32234±2.31071 | 39.05619±4.05386 | 38.68197±3.67216 | 25.16078±0.74183 | 29.685±1.18343 | 40.555±6.25333 | 39.65714±7.54568 | 36.00125±9.31478 | 20.95±2.1764 | 1.000 | 0.955 | 1.058 | 1.015 | 0.931 | 0.833 |
| PC 39:4 | 0.01789±0.00579 | 0.02926±0.00352 | 0.02817±0.00284 | 0.02992±0.00407 | 0.02514±0.0015 | 0.01869±0.00124 | 0.02637±0.00498 | 0.01945±0.00208 | 0.01655±0.00274 | 0.0144±0.00365 | 0.00863±0.00107 | 1.000 | 0.901 | 0.691 | 0.553 | 0.573 | 0.462 |
| PC 39:6 | 0.02446±0.0039 | 0.02202±0.00175 | 0.02847±0.00373 | 0.0298±0.00147 | 0.02547±0.0061 | 0.02208±0.00233 | 0.02747±0.00381 | 0.03148±0.00717 | 0.03066±0.00721 | 0.03322±0.00551 | 0.02199±0.00361 | 1.000 | 1.248 | 1.106 | 1.029 | 1.304 | 0.996 |
| PC 40:4 | 0.03128±0.00268 | 0.02727±0.00267 | 0.03745±0.00664 | 0.03427±0.00378 | 0.03394±0.00461 | 0.02717±0.00431 | 0.01634±0.00472 | 0.01303±0.00254 | 0.01314±0.00285 | 0.01104±0.00166 | 0.01107±0.0025 | 1.000 | 0.599 | 0.348 | 0.384 | 0.325 | 0.407 |
| PC 40:6 | 4.993±0.74382 | 4.5251±0.37148 | 5.45073±0.42808 | 5.68088±0.78338 | 4.72522±0.82425 | 3.93123±0.13149 | 4.7445±0.33334 | 5.18233±0.67707 | 5.94671±1.0473 | 5.64738±1.24417 | 4.163±0.39839 | 1.000 | 1.048 | 0.951 | 1.047 | 1.195 | 1.059 |
| PC 40:7 | 0.71801±0.09184 | 0.68052±0.05626 | 0.823±0.06857 | 0.84882±0.08236 | 0.75798±0.12302 | 0.31026±0.01761 | 0.69467±0.04035 | 0.86388±0.1249 | 0.8514±0.19558 | 0.61738±0.20157 | 0.29743±0.08781 | 1.000 | 1.021 | 1.050 | 1.003 | 0.815 | 0.959 |
| PC 40:8 | 0.2182±0.01473 | 0.2007±0.01024 | 0.23276±0.01956 | 0.22783±0.02407 | 0.2175±0.02088 | 0.15691±0.00366 | 0.21795±0.02789 | 0.26615±0.02659 | 0.2711±0.07451 | 0.20081±0.058 | 0.1089±0.04636 | 1.000 | 1.086 | 1.144 | 1.190 | 0.923 | 0.694 |
| PC 41:6 | 0.00783±0.00056 | 0.00738±0.00047 | 0.008±0.0009 | 0.00784±0.00078 | 0.00698±0.00183 | 0.00604±0.0002 | 0.00919±0.00125 | 0.00839±0.00169 | 0.00858±0.00157 | 0.00793±0.00181 | 0.00494±0.00108 | 1.000 | 1.245 | 1.049 | 1.094 | 1.136 | 0.818 |
| LPC 14:0 | 0.00616±0.0036 | 0.00673±0.00522 | 0.00535±0.00185 | 0.00695±0.00555 | 0.00292±0.00061 | 0.00591±0.00323 | 0.00733±0.00119 | 0.00858±0.00243 | 0.01057±0.00963 | 0.02105±0.01693 | 0.00674±0.00246 | 1.000 | 1.090 | 1.604 | 1.521 | 7.203 | 1.140 |
| LPC 16:0 | 10.59±1.03348 | 10.45901±1.64718 | 10.9861±0.47103 | 11.12786±0.96495 | 10.81456±1.53462 | 10.49984±1.00689 | 10.33833±1.58702 | 9.20033±2.24393 | 7.98343±1.76049 | 10.62188±1.38047 | 7.64733±2.46516 | 1.000 | 0.988 | 0.837 | 0.717 | 0.982 | 0.728 |
| LPC 16:1 | 0.04824±0.00615 | 0.04321±0.00469 | 0.04011±0.00589 | 0.04119±0.00575 | 0.03464±0.0105 | 0.03268±0.00228 | 0.04167±0.00688 | 0.03689±0.0065 | 0.04115±0.01225 | 0.03033±0.00673 | 0.02654±0.00505 | 1.000 | 0.964 | 0.920 | 0.999 | 0.876 | 0.812 |
| LPC 18:0 | 3.97999±0.49852 | 3.75851±0.29314 | 3.52983±0.28179 | 4.13248±0.33432 | 3.61427±0.35806 | 4.21335±0.25659 | 3.83833±0.28644 | 3.655±0.70215 | 3.11243±0.48681 | 3.9715±0.59124 | 2.972±0.82632 | 1.000 | 1.021 | 1.035 | 0.753 | 1.099 | 0.705 |
| LPC 18:1 | 0.84345±0.11594 | 0.77533±0.13593 | 0.83666±0.12038 | 0.77872±0.13767 | 0.7041±0.04958 | 0.66412±0.06824 | 0.80603±0.09982 | 0.94718±0.31927 | 0.76104±0.16593 | 0.85924±0.10711 | 0.58557±0.22883 | 1.000 | 1.039 | 1.132 | 0.977 | 1.220 | 0.882 |
| LPC 18:2 | 5.48056±0.9665 | 5.16924±0.74058 | 5.07578±0.71885 | 5.20967±0.90732 | 4.68055±0.47043 | 6.77296±1.21087 | 5.66733±0.60408 | 7.68517±2.61581 | 5.67714±1.24603 | 5.77963±1.48019 | 4.00433±1.22919 | 1.000 | 1.096 | 1.514 | 1.090 | 1.235 | 0.591 |
| LPC 18:3 | 0.06754±0.01333 | 0.05546±0.01177 | 0.06228±0.01142 | 0.05524±0.01206 | 0.04439±0.01318 | 0.0503±0.00829 | 0.06839±0.02293 | 0.10658±0.0381 | 0.05839±0.0192 | 0.04219±0.0201 | 0.01884±0.00985 | 1.000 | 1.233 | 1.711 | 1.057 | 0.950 | 0.375 |
| LPC 19:0 | 0.04088±0.00348 | 0.0385±0.00382 | 0.03568±0.00228 | 0.04012±0.00372 | 0.03575±0.0042 | 0.04182±0.00503 | 0.04879±0.00606 | 0.0394±0.0077 | 0.02714±0.00629 | 0.03266±0.00573 | 0.02834±0.01449 | 1.000 | 1.267 | 1.104 | 0.677 | 0.914 | 0.678 |
| LPC 20:0 | 0.05205±0.00867 | 0.05473±0.00291 | 0.0457±0.00263 | 0.04706±0.00786 | 0.0385±0.0054 | 0.07995±0.00777 | 0.0301±0.00663 | 0.02775±0.00247 | 0.01787±0.00478 | 0.01859±0.00489 | 0.02044±0.0117 | 1.000 | 0.550 | 0.607 | 0.380 | 0.483 | 0.256 |
| LPC 20:1 | 0.0106±0.00204 | 0.01017±0.00118 | 0.00987±0.00068 | 0.0096±0.0016 | 0.00849±0.0024 | 0.01263±0.00034 | 0.01147±0.00113 | 0.00842±0.00226 | 0.00764±0.00171 | 0.00944±0.00173 | 0.00813±0.00284 | 1.000 | 1.128 | 0.854 | 0.796 | 1.112 | 0.644 |
| LPC 20:2 | 0.0075±0.00158 | 0.00768±0.00087 | 0.00818±0.00136 | 0.0075±0.00168 | 0.0068±0.00155 | 0.01757±0.00147 | 0.00523±0.00088 | 0.00539±0.00223 | 0.00479±0.00093 | 0.00791±0.0023 | 0.00721±0.00371 | 1.000 | 0.681 | 0.659 | 0.638 | 1.163 | 0.410 |
| LPC 20:4 | 1.1898±0.15902 | 1.08274±0.11621 | 1.01654±0.14539 | 1.10464±0.17326 | 0.9123±0.08163 | 0.80595±0.06418 | 1.549±0.24962 | 1.688±0.48455 | 1.17407±0.14756 | 1.11144±0.18052 | 0.72513±0.16417 | 1.000 | 1.431 | 1.661 | 1.063 | 1.218 | 0.900 |
| LPC 22:0 | 0.0083±0.00093 | 0.00841±0.00024 | 0.0076±0.00058 | 0.00925±0.00128 | 0.00831±0.00138 | 0.01091±0.00099 | 0.00769±0.00117 | 0.01006±0.00181 | 0.00779±0.00158 | 0.01021±0.00152 | 0.00743±0.00343 | 1.000 | 0.914 | 1.324 | 0.843 | 1.229 | 0.681 |
| LPC 22:6 | 1.15421±0.20896 | 0.94444±0.14324 | 1.0535±0.13542 | 1.08303±0.16013 | 0.77387±0.17371 | 1.04845±0.00429 | 1.18742±0.36352 | 1.58318±0.59016 | 1.10396±0.26834 | 1.29463±0.12303 | 0.94673±0.36525 | 1.000 | 1.257 | 1.503 | 1.019 | 1.673 | 0.903 |
| LPC 24:0 | 0.01068±0.00132 | 0.0105±0.00036 | 0.00939±0.00072 | 0.01197±0.00209 | 0.01143±0.00201 | 0.01264±0.00165 | 0.01094±0.00175 | 0.0142±0.00321 | 0.01239±0.00255 | 0.01514±0.0025 | 0.01172±0.00479 | 1.000 | 1.042 | 1.512 | 1.035 | 1.325 | 0.927 |
| LPC 24:1 | 0.00234±0.0003 | 0.00251±0.00017 | 0.00219±0.00026 | 0.0025±0.00046 | 0.00233±0.00033 | 0.00258±0.00037 | 0.0024±0.00031 | 0.00281±0.00073 | 0.00205±0.00045 | 0.00276±0.00029 | 0.00224±0.00075 | 1.000 | 0.956 | 1.282 | 0.819 | 1.184 | 0.866 |
| PC O-34:1 | 0.03159±0.00353 | 0.02787±0.00205 | 0.02953±0.00234 | 0.02963±0.00256 | 0.02976±0.00264 | 0.03362±0.00131 | 0.03636±0.00574 | 0.03738±0.00474 | 0.03551±0.00628 | 0.04959±0.00719 | 0.0501±0.01935 | 1.000 | 1.305 | 1.266 | 1.199 | 1.666 | 1.491 |
| PC O-34:2 | 0.03124±0.00314 | 0.02787±0.00214 | 0.03222±0.00356 | 0.02973±0.00455 | 0.03138±0.00419 | 0.06637±0.0038 | 0.02591±0.00467 | 0.02565±0.00232 | 0.02622±0.00528 | 0.03155±0.00749 | 0.03375±0.01458 | 1.000 | 0.929 | 0.796 | 0.882 | 1.005 | 0.508 |
| PC O-34:3 | 0.01516±0.00145 | 0.01365±0.00165 | 0.01476±0.00289 | 0.0131±0.00177 | 0.01453±0.0035 | 0.02668±0.00327 | 0.01076±0.00154 | 0.01078±0.00203 | 0.01141±0.00298 | 0.01114±0.00314 | 0.01044±0.0057 | 1.000 | 0.788 | 0.730 | 0.871 | 0.767 | 0.391 |
| PC O-36:2 | 0.01784±0.00272 | 0.01566±0.00235 | 0.01556±0.0021 | 0.01261±0.00133 | 0.01217±0.00192 | 0.01789±0.00213 | 0.01866±0.0046 | 0.01627±0.00389 | 0.01655±0.00451 | 0.0161±0.00466 | 0.01775±0.0122 | 1.000 | 1.192 | 1.045 | 1.312 | 1.323 | 0.992 |
| PC O-36:4 | 0.26664±0.02667 | 0.23823±0.01749 | 0.24818±0.02381 | 0.27127±0.02686 | 0.29865±0.02636 | 0.37417±0.02331 | 0.29583±0.02577 | 0.26758±0.02291 | 0.28166±0.05037 | 0.38075±0.05289 | 0.33983±0.08745 | 1.000 | 1.242 | 1.078 | 1.038 | 1.275 | 0.908 |
| PC O-38:4 | 0.02612±0.00246 | 0.02335±0.00353 | 0.02425±0.00263 | 0.02332±0.00151 | 0.02316±0.00196 | 0.02811±0.00258 | 0.03354±0.0065 | 0.03085±0.00466 | 0.03079±0.0068 | 0.04443±0.00949 | 0.03836±0.01615 | 1.000 | 1.436 | 1.272 | 1.320 | 1.918 | 1.364 |
| PC O-38:6 | 0.03375±0.00391 | 0.02998±0.00291 | 0.03233±0.00318 | 0.03518±0.00215 | 0.03796±0.00557 | 0.04047±0.00256 | 0.03603±0.00445 | 0.034±0.00631 | 0.03519±0.00829 | 0.05833±0.01269 | 0.07444±0.01883 | 1.000 | 1.202 | 1.052 | 1.000 | 1.537 | 1.840 |
| LPC O-16:0 | 0.0067±0.00092 | 0.00621±0.00047 | 0.00671±0.00037 | 0.0077±0.00056 | 0.00802±0.00063 | 0.01085±0.0007 | 0.00766±0.00099 | 0.00849±0.00246 | 0.01009±0.00184 | 0.01265±0.00167 | 0.01513±0.00327 | 1.000 | 1.233 | 1.265 | 1.311 | 1.578 | 1.394 |
|  |  |  |  |  |  |  |  |  |  |  |  |  |  |  |  |  |  |
| PE 34:0 | 0.02866±0.00123 | 0.02796±0.00158 | 0.0257±0.00351 | 0.02605±0.00151 | 0.02457±0.00302 | 0.02363±0.003 | 0.0354±0.01056 | 0.04576±0.00941 | 0.03864±0.00763 | 0.04807±0.01171 | 0.041±0.022 | 1.000 | 1.266 | 1.780 | 1.483 | 1.957 | 1.740 |
| PE 34:2 | 0.189±0.04741 | 0.17611±0.02152 | 0.21343±0.06274 | 0.19529±0.03893 | 0.14515±0.01815 | 0.18978±0.04474 | 0.17898±0.07262 | 0.94652±0.3393 | 1.04994±0.41391 | 0.35824±0.09526 | 0.233±0.075 | 1.000 | 1.016 | 4.435 | 5.376 | 2.468 | 1.227 |
| PE 36:3 | 0.08934±0.03009 | 0.08042±0.01968 | 0.09084±0.01995 | 0.08665±0.02545 | 0.07283±0.01483 | 0.09533±0.03863 | 0.0787±0.03138 | 0.19315±0.033 | 0.33443±0.12917 | 0.13131±0.02653 | 0.091±0.043 | 1.000 | 0.979 | 2.127 | 3.859 | 1.802 | 0.954 |
| PE 36:4 | 0.1792±0.03709 | 0.14928±0.00926 | 0.2283±0.06398 | 0.24696±0.04744 | 0.19323±0.02576 | 0.1553±0.02614 | 0.15352±0.05805 | 0.39103±0.13236 | 0.57536±0.19392 | 0.38809±0.07225 | 0.251±0.125 | 1.000 | 1.028 | 1.713 | 2.330 | 2.008 | 1.617 |
| PE 38:4 | 0.47173±0.11709 | 0.42337±0.01494 | 0.53599±0.10965 | 0.59549±0.08922 | 0.48459±0.08498 | 0.38574±0.07303 | 0.49717±0.18734 | 1.55850±0.32264 | 3.55960±0.72663 | 1.84538±0.71786 | 0.973±0.275 | 1.000 | 1.174 | 2.908 | 5.978 | 3.807 | 2.523 |
| PE 38:6 | 0.91006±0.21877 | 0.82218±0.03632 | 1.09494±0.22694 | 1.21775±0.18441 | 1.01686±0.22501 | 0.57275±0.11029 | 0.83115±0.32212 | 2.65575±0.40388 | 3.20020±0.37550 | 2.365±0.80415 | 1.406±0.655 | 1.000 | 1.011 | 2.425 | 2.628 | 2.326 | 2.454 |
| PE 40:7 | 0.1661±0.04522 | 0.14573±0.01426 | 0.20354±0.04093 | 0.23245±0.02304 | 0.2112±0.05211 | 0.08219±0.02733 | 0.17109±0.082 | 0.30462±0.08915 | 0.34153±0.0821 | 0.31121±0.09555 | 0.176±0.093 | 1.000 | 1.174 | 1.497 | 1.469 | 1.473 | 2.135 |
| LPE 16:0 | 0.0775±0.01331 | 0.07996±0.00956 | 0.09868±0.01446 | 0.09308±0.01474 | 0.08348±0.01316 | 0.08169±0.00797 | 0.09375±0.04393 | 0.14812±0.06121 | 0.16173±0.0527 | 0.14071±0.01975 | 0.108±0.076 | 1.000 | 1.172 | 1.501 | 1.738 | 1.685 | 1.320 |
| LPE 18:0 | 0.06908±0.01021 | 0.07333±0.012 | 0.08657±0.01284 | 0.08668±0.00924 | 0.07794±0.0139 | 0.10345±0.0123 | 0.07598±0.02838 | 0.14956±0.06033 | 0.19635±0.06344 | 0.16659±0.0328 | 0.126±0.078 | 1.000 | 1.036 | 1.728 | 2.266 | 2.138 | 1.215 |
| LPE 18:1 | 0.02866±0.00567 | 0.02659±0.00412 | 0.03439±0.00775 | 0.03158±0.0059 | 0.02599±0.00335 | 0.03804±0.00579 | 0.03133±0.00751 | 0.0574±0.02199 | 0.0496±0.01582 | 0.04865±0.01022 | 0.035±0.025 | 1.000 | 1.178 | 1.669 | 1.571 | 1.872 | 0.912 |
| LPE 18:2 | 0.08574±0.01878 | 0.07898±0.01355 | 0.08918±0.02138 | 0.06782±0.01674 | 0.05071±0.00591 | 0.15953±0.0286 | 0.10175±0.05999 | 0.24112±0.11458 | 0.1753±0.11863 | 0.12027±0.02633 | 0.128±0.09 | 1.000 | 1.289 | 2.704 | 2.585 | 2.371 | 0.804 |
| LPE 20:4 | 0.10651±0.01449 | 0.10351±0.02455 | 0.0987±0.015 | 0.10017±0.00539 | 0.07023±0.00745 | 0.10428±0.01353 | 0.12252±0.06385 | 0.22393±0.12596 | 0.24299±0.21492 | 0.12508±0.06552 | 0.144±0.111 | 1.000 | 1.184 | 2.268 | 2.426 | 1.781 | 1.378 |
| LPE 22:6 | 0.24349±0.02163 | 0.2324±0.05077 | 0.20588±0.03868 | 0.21791±0.02265 | 0.13829±0.01963 | 0.23078±0.02391 | 0.29871±0.21476 | 0.72243±0.44905 | 0.61646±0.76369 | 0.2815±0.17572 | 0.346±0.277 | 1.000 | 1.285 | 3.509 | 2.829 | 2.036 | 1.498 |
| PE O-36:5 | 0.08311±0.01119 | 0.07126±0.00832 | 0.0647±0.01028 | 0.06279±0.01163 | 0.06524±0.01037 | 0.08515±0.01279 | 0.09485±0.0343 | 0.09142±0.02696 | 0.06737±0.01682 | 0.11781±0.04687 | 0.093±0.066 | 1.000 | 1.331 | 1.413 | 1.073 | 1.806 | 1.092 |
| PE O-38:5 | 0.07578±0.00777 | 0.0673±0.0114 | 0.05993±0.0104 | 0.05722±0.01244 | 0.06599±0.01132 | 0.10905±0.01456 | 0.0875±0.02369 | 0.07805±0.02282 | 0.06347±0.01686 | 0.12824±0.04455 | 0.141±0.111 | 1.000 | 1.300 | 1.302 | 1.109 | 1.944 | 1.294 |
| PE O-38:7 | 0.18261±0.01744 | 0.15955±0.00866 | 0.15561±0.02181 | 0.14476±0.02133 | 0.14665±0.02855 | 0.16517±0.02896 | 0.21682±0.06541 | 0.22272±0.05596 | 0.15839±0.04179 | 0.22883±0.06523 | 0.18±0.121 | 1.000 | 1.359 | 1.431 | 1.094 | 1.560 | 1.088 |
| PE O-40:6 | 0.15973±0.01878 | 0.14285±0.02214 | 0.13456±0.01316 | 0.11949±0.01884 | 0.12651±0.02483 | 0.136±0.02315 | 0.24825±0.02959 | 0.1902±0.0312 | 0.11789±0.0315 | 0.16731±0.03669 | 0.14±0.106 | 1.000 | 1.738 | 1.414 | 0.987 | 1.323 | 1.032 |
| PE O-40:7 | 0.15329±0.01262 | 0.13602±0.01466 | 0.13356±0.01461 | 0.12215±0.01525 | 0.13227±0.02424 | 0.17621±0.02999 | 0.18775±0.06051 | 0.18195±0.05683 | 0.14497±0.04481 | 0.213±0.04853 | 0.233±0.169 | 1.000 | 1.380 | 1.362 | 1.187 | 1.610 | 1.323 |
