# Supplementary figures and images for: Liquid chromatography mass spectrometry-based profiling of phosphatidylcholine and phosphatidylethanolamine in the plasma and liver of acetaminophen-induced liver injured mice
Source: Lipids Health Dis. 2017 Aug 14;16:153. doi: 10.1186/s12944-017-0540-4 (PMC5556666; doi:10.1186/s12944-017-0540-4)

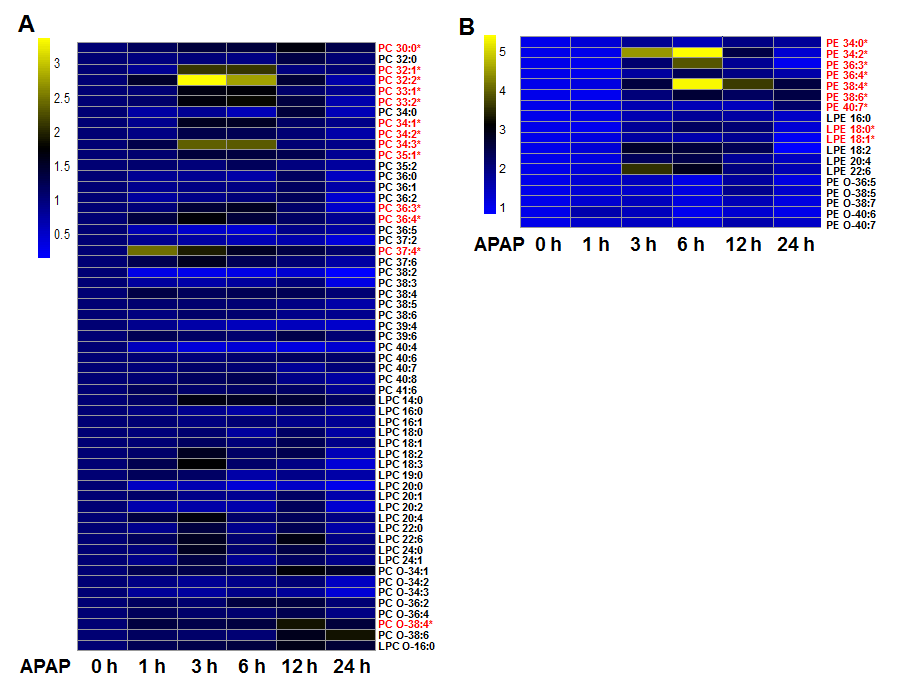

Supplement: Supplementary file 2 — Heat map for PC and PE profiles in the plasma of APAP-treated mice. The mean of the concentration of PC(A)/PE(B) in plasma of APAP-treated mice at indicated time point (n = 6~8 for each time point) were normalized to the saline-treated group at the same time point. The color of each section is proportional to the relative concentration of phospholipids as shown in the color bar (blue-black-yellow colors correspond to low-moderate-high levels respectively). Rows: phospholipid species; columns: samples at indicated time point. (TIFF 48 kb) [file 12944_2017_540_MOESM2_ESM.tif]

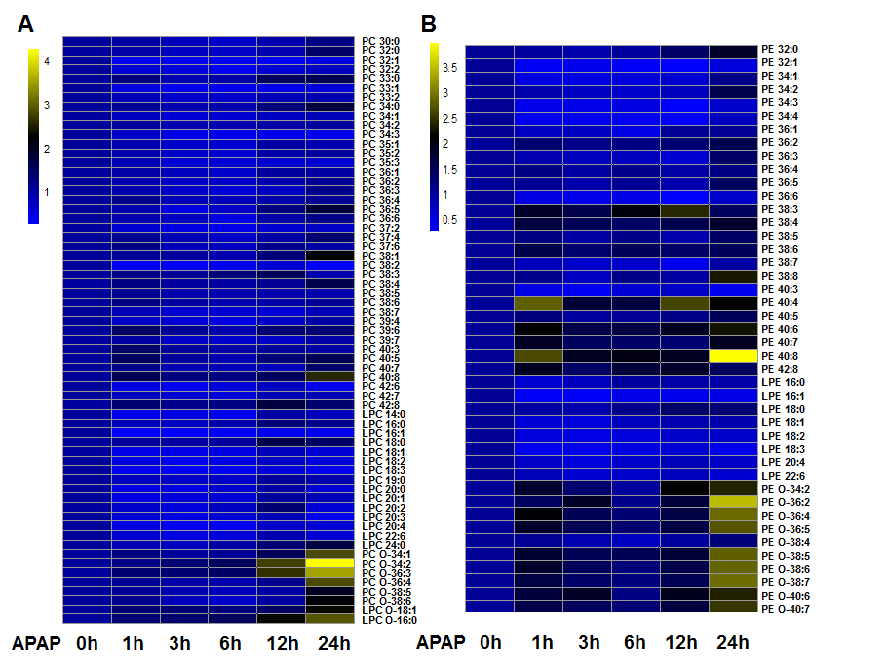

Supplement: Supplementary file 3 — Heat map for PC and PE profiles in the livers of APAP-treated mice. The mean of the concentration of PC(A)/PE(B) in livers of APAP-treated mice at indicated time point (n = 6~8 for each time point) were normalized to the saline-treated group at the same time point. The color of each section is proportional to the relative concentration of phospholipids as shown in the color bar (blue-black-yellow colors correspond to low-moderate-high levels respectively). Rows: phospholipid species; columns: samples at indicated time point. (TIFF 57 kb) [file 12944_2017_540_MOESM3_ESM.tif]
