# Supplementary material for: Liquid chromatography mass spectrometry-based profiling of phosphatidylcholine and phosphatidylethanolamine in the plasma and liver of acetaminophen-induced liver injured mice
Source: Lipids Health Dis. 2017 Aug 14;16:153. doi: 10.1186/s12944-017-0540-4 (PMC5556666; doi:10.1186/s12944-017-0540-4)
Supplement: Supplementary file 4 — The original data of PC/PE concentrations and the fold changes of APAP-treated mice compared with saline-treated mice at indicated time points in liver. The original data were presented as the mean ± SD. The lipid species with statistical significance were labeled with red. (DOCX 39 kb) [file 12944_2017_540_MOESM4_ESM.docx]

|  | N/A | Saline | | | | | APAP (300 mg/kg) | | | | | APAP/Saline | | | | | |
| --- | --- | --- | --- | --- | --- | --- | --- | --- | --- | --- | --- | --- | --- | --- | --- | --- | --- |
| Liver PC/PE | 0 h | 1 h | 3 h | 6 h | 12 h | 24 h | 1 h | 3 h | 6 h | 12 h | 24 h | 0 h | 1 h | 3 h | 6 h | 12 h | 24 h |
| PC 30:0 | 0.19811±0.04227 | 0.18048±0.03333 | 0.17909±0.02503 | 0.22266±0.01135 | 0.21281±0.01663 | 0.14701±0.01441 | 0.17339±0.01088 | 0.13867±0.00983 | 0.14611±0.02996 | 0.19111±0.02936 | 0.17605±0.03774 | 1.000 | 0.961 | 0.774 | 0.656 | 0.898 | 1.197 |
| PC 32:0 | 4.77401±0.78722 | 4.86833±1.13563 | 5.21365±0.49075 | 5.76978±0.44472 | 6.0175±0.68215 | 3.21438±0.43953 | 4.49924±0.59178 | 3.88915±0.42985 | 3.92841±0.66526 | 5.09823±0.65695 | 4.55457±0.32103 | 1.000 | 0.924 | 0.746 | 0.681 | 0.847 | 1.417 |
| PC 32:1 | 3.31555±0.82978 | 4.27514±0.78333 | 3.86593±0.31589 | 4.27244±0.76042 | 4.24694±1.067 | 3.1202±0.2841 | 1.74552±0.1605 | 1.43194±0.14003 | 1.64483±0.25545 | 1.31676±0.30625 | 1.34977±0.56049 | 1.000 | 0.408 | 0.370 | 0.385 | 0.310 | 0.433 |
| PC 32:2 | 0.58457±0.12822 | 0.84817±0.12587 | 0.74784±0.10767 | 1.18566±0.25252 | 0.83896±0.1567 | 0.45931±0.06163 | 0.50839±0.0761 | 0.38552±0.04564 | 0.47377±0.08112 | 0.48075±0.15312 | 0.33347±0.09591 | 1.000 | 0.599 | 0.516 | 0.400 | 0.573 | 0.726 |
| PC 33:0 | 0.07344±0.01564 | 0.06663±0.01598 | 0.07677±0.0261 | 0.07606±0.03067 | 0.06405±0.02747 | 0.05669±0.01418 | 0.0829±0.01156 | 0.06423±0.01974 | 0.0715±0.01432 | 0.09455±0.00761 | 0.08987±0.00916 | 1.000 | 1.244 | 0.837 | 0.940 | 1.476 | 1.585 |
| PC 33:1 | 0.5032±0.07281 | 0.70117±0.11362 | 0.67711±0.09536 | 0.75263±0.087 | 0.61617±0.10362 | 0.50063±0.0533 | 0.34986±0.04049 | 0.25151±0.02647 | 0.27834±0.03157 | 0.26638±0.04793 | 0.26307±0.07547 | 1.000 | 0.499 | 0.371 | 0.370 | 0.432 | 0.525 |
| PC 33:2 | 0.75787±0.11183 | 0.98762±0.102 | 1.0647±0.15258 | 1.17781±0.19707 | 0.88705±0.05635 | 0.7274±0.14861 | 0.80081±0.1099 | 0.68298±0.1204 | 0.76794±0.06958 | 0.71166±0.05319 | 0.60871±0.04818 | 1.000 | 0.811 | 0.641 | 0.652 | 0.802 | 0.837 |
| PC 34:0 | 0.49772±0.07654 | 0.40404±0.08281 | 0.44624±0.04265 | 0.49546±0.07057 | 0.48239±0.02408 | 0.40152±0.06378 | 0.42736±0.02704 | 0.38154±0.02666 | 0.44006±0.07428 | 0.61997±0.06295 | 0.69582±0.13538 | 1.000 | 1.058 | 0.855 | 0.888 | 1.285 | 1.733 |
| PC 34:1 | 36.27104±4.47894 | 40.50633±8.91739 | 37.34252±3.49447 | 42.38542±2.40224 | 39.7557±5.3686 | 38.0292±6.63198 | 31.48085±3.29382 | 21.45523±2.90555 | 25.7991±4.05444 | 29.52806±4.50404 | 31.16748±5.92748 | 1.000 | 0.777 | 0.575 | 0.609 | 0.743 | 0.820 |
| PC 34:2 | 97.4237±7.11374 | 101.64079±16.56999 | 106.508±8.41307 | 115.91531±9.31308 | 108.5824±8.72404 | 82.17232±7.24227 | 102.33681±14.14568 | 89.49017±4.75152 | 99.77334±13.42132 | 100.8526±12.5224 | 82.81461±5.00526 | 1.000 | 1.007 | 0.840 | 0.861 | 0.929 | 1.008 |
| PC 34:3 | 3.78655±0.60146 | 5.53823±0.43789 | 5.88591±0.82512 | 5.73074±0.97218 | 5.17517±0.51479 | 3.76076±0.52621 | 3.7536±0.39519 | 2.55408±0.08047 | 2.38097±0.29686 | 2.13745±0.40676 | 1.56932±0.50339 | 1.000 | 0.678 | 0.434 | 0.415 | 0.413 | 0.417 |
| PC 35:1 | 0.31562±0.0528 | 0.31186±0.03383 | 0.32273±0.03934 | 0.38765±0.02775 | 0.2939±0.05118 | 0.35154±0.07265 | 0.27999±0.03624 | 0.20816±0.02712 | 0.23409±0.02795 | 0.28076±0.03543 | 0.29601±0.03992 | 1.000 | 0.898 | 0.645 | 0.604 | 0.955 | 0.842 |
| PC 35:2 | 1.08787±0.15429 | 1.28982±0.13431 | 1.33129±0.18339 | 1.56614±0.2402 | 1.14815±0.1481 | 0.95317±0.20138 | 1.14859±0.21775 | 0.8857±0.1178 | 0.98085±0.10844 | 1.05989±0.12333 | 0.94645±0.10793 | 1.000 | 0.891 | 0.665 | 0.626 | 0.923 | 0.993 |
| PC 35:3 | 0.40037±0.04953 | 0.46384±0.05272 | 0.48085±0.04665 | 0.53898±0.07719 | 0.4181±0.04359 | 0.33422±0.07886 | 0.37175±0.04393 | 0.2812±0.0352 | 0.29857±0.03643 | 0.25207±0.04728 | 0.19954±0.03009 | 1.000 | 0.801 | 0.585 | 0.554 | 0.603 | 0.597 |
| PC 36:1 | 2.63054±0.23132 | 2.36702±0.41588 | 2.25433±0.17903 | 2.97487±0.27793 | 2.2393±0.21723 | 2.42696±0.25887 | 2.17265±0.21572 | 1.71404±0.13018 | 1.94258±0.23122 | 2.27572±0.17545 | 2.32768±0.18037 | 1.000 | 0.918 | 0.760 | 0.653 | 1.016 | 0.959 |
| PC 36:2 | 37.36102±4.83156 | 39.09748±7.2952 | 40.554±4.17433 | 49.18979±6.24181 | 38.75787±2.16123 | 28.62869±4.72041 | 33.62747±8.01864 | 28.3212±3.30424 | 34.74163±4.75564 | 34.93001±5.71658 | 31.63385±3.89315 | 1.000 | 0.860 | 0.698 | 0.706 | 0.901 | 1.105 |
| PC 36:3 | 11.21039±2.28349 | 14.2834±1.49337 | 13.95546±0.75638 | 19.70682±2.99609 | 13.59643±2.06115 | 6.57879±2.31169 | 12.08112±1.78879 | 9.90063±1.81184 | 11.37344±1.3748 | 9.99562±1.41114 | 7.33965±1.24807 | 1.000 | 0.846 | 0.709 | 0.577 | 0.735 | 1.116 |
| PC 36:4 | 58.25786±9.12451 | 49.19085±9.24944 | 55.95704±5.21316 | 58.97944±2.78823 | 59.07127±5.86622 | 38.27669±6.85813 | 55.27105±5.86652 | 46.43±2.79437 | 46.31095±4.03638 | 52.14866±5.75854 | 42.209±5.06304 | 1.000 | 1.124 | 0.830 | 0.785 | 0.883 | 1.103 |
| PC 36:5 | 1.81467±0.44186 | 0.96022±0.18087 | 1.35673±0.10924 | 1.34078±0.11249 | 1.24208±0.07022 | 0.55937±0.12295 | 0.8372±0.21776 | 0.64898±0.08952 | 0.94183±0.24901 | 1.54018±0.29181 | 0.98289±0.34516 | 1.000 | 0.872 | 0.478 | 0.702 | 1.240 | 1.757 |
| PC 36:6 | 0.13353±0.00409 | 0.10908±0.01412 | 0.10158±0.01163 | 0.16427±0.02034 | 0.12768±0.01128 | 0.06708±0.0074 | 0.0984±0.01346 | 0.07614±0.00741 | 0.07873±0.01795 | 0.12034±0.02913 | 0.07722±0.02528 | 1.000 | 0.902 | 0.750 | 0.479 | 0.942 | 1.151 |
| PC 37:2 | 0.31618±0.06031 | 0.4527±0.04398 | 0.46991±0.05781 | 0.55922±0.08607 | 0.35992±0.06128 | 0.27226±0.10098 | 0.27052±0.06357 | 0.22779±0.0285 | 0.24081±0.01764 | 0.2244±0.04701 | 0.19215±0.02452 | 1.000 | 0.598 | 0.485 | 0.431 | 0.623 | 0.706 |
| PC 37:4 | 0.65094±0.14625 | 0.5923±0.08341 | 0.64199±0.10562 | 0.7024±0.05191 | 0.55978±0.07962 | 0.29494±0.0507 | 0.64238±0.13467 | 0.51263±0.07753 | 0.48148±0.06244 | 0.55282±0.08881 | 0.40507±0.04058 | 1.000 | 1.085 | 0.798 | 0.685 | 0.988 | 1.373 |
| PC 37:6 | 0.18193±0.00964 | 0.1339±0.00868 | 0.16078±0.0282 | 0.16299±0.01739 | 0.13953±0.01715 | 0.12848±0.03759 | 0.16019±0.03681 | 0.12559±0.01529 | 0.12019±0.02221 | 0.15537±0.01535 | 0.11972±0.02858 | 1.000 | 1.196 | 0.781 | 0.737 | 1.114 | 0.932 |
| PC 38:1 | 0.05836±0.00946 | 0.06051±0.01065 | 0.05351±0.01007 | 0.0622±0.00962 | 0.04666±0.00571 | 0.02644±0.00727 | 0.06391±0.00621 | 0.05384±0.00951 | 0.05368±0.00761 | 0.06042±0.00949 | 0.05925±0.02259 | 1.000 | 1.056 | 1.006 | 0.863 | 1.295 | 2.240 |
| PC 38:2 | 0.5232±0.11553 | 0.50228±0.07941 | 0.54372±0.08343 | 0.49288±0.03354 | 0.33058±0.007 | 0.93355±0.30885 | 0.18021±0.03512 | 0.1722±0.02571 | 0.19644±0.04015 | 0.20126±0.02152 | 0.24976±0.05155 | 1.000 | 0.359 | 0.317 | 0.399 | 0.609 | 0.268 |
| PC 38:3 | 2.43632±0.51141 | 1.02499±0.1729 | 1.03109±0.13618 | 0.9589±0.19746 | 0.84654±0.16929 | 2.00735±0.37975 | 1.18636±0.18268 | 1.07971±0.11253 | 1.25262±0.11992 | 1.30091±0.13294 | 1.77295±0.47653 | 1.000 | 1.157 | 1.047 | 1.306 | 1.537 | 0.883 |
| PC 38:4 | 27.15723±2.75564 | 21.18733±4.10857 | 21.71471±3.19974 | 23.99318±2.93367 | 23.65159±2.54525 | 11.83997±2.23286 | 25.86631±2.05834 | 21.9517±3.80215 | 22.8933±1.98294 | 25.6885±2.93146 | 19.06826±2.68706 | 1.000 | 1.221 | 1.011 | 0.954 | 1.086 | 1.610 |
| PC 38:5 | 7.15043±1.52544 | 5.77052±0.90111 | 6.17158±0.47663 | 6.34339±0.4562 | 6.51289±0.96114 | 4.83609±0.09095 | 6.36969±0.85609 | 5.50241±0.27989 | 5.36713±0.73581 | 5.69669±0.75211 | 4.24742±0.79649 | 1.000 | 1.104 | 0.892 | 0.846 | 0.875 | 0.878 |
| PC 38:6 | 63.16856±3.02091 | 51.16068±7.41918 | 55.67119±7.26855 | 59.8092±5.21569 | 64.8495±8.80922 | 48.39006±3.11319 | 62.72292±11.56913 | 53.80902±3.71438 | 50.62022±8.35123 | 61.4079±9.98071 | 50.66933±4.41606 | 1.000 | 1.226 | 0.967 | 0.846 | 0.947 | 1.047 |
| PC 38:7 | 0.94868±0.13545 | 0.71358±0.09465 | 0.74237±0.07086 | 0.81248±0.13273 | 0.75069±0.08386 | 0.41072±0.07314 | 0.59261±0.10912 | 0.46059±0.05455 | 0.44979±0.06412 | 0.43596±0.08624 | 0.32966±0.11776 | 1.000 | 0.830 | 0.620 | 0.554 | 0.581 | 0.803 |
| PC 39:4 | 0.17243±0.04074 | 0.22616±0.03089 | 0.22933±0.03652 | 0.2478±0.02866 | 0.17946±0.04408 | 0.09856±0.01923 | 0.1564±0.03499 | 0.12689±0.01404 | 0.12183±0.01547 | 0.12508±0.02548 | 0.09011±0.01473 | 1.000 | 0.692 | 0.553 | 0.492 | 0.697 | 0.914 |
| PC 39:6 | 0.50679±0.06265 | 0.32344±0.02723 | 0.37248±0.03922 | 0.39282±0.03419 | 0.3531±0.06086 | 0.31231±0.0671 | 0.46599±0.07889 | 0.38705±0.04121 | 0.35648±0.05393 | 0.46894±0.05872 | 0.41405±0.07326 | 1.000 | 1.441 | 1.039 | 0.907 | 1.328 | 1.326 |
| PC 39:7 | 0.08009±0.01145 | 0.04995±0.0042 | 0.05604±0.00542 | 0.05384±0.00504 | 0.0515±0.00581 | 0.0373±0.00783 | 0.04925±0.00939 | 0.03788±0.00643 | 0.03483±0.00459 | 0.035±0.00399 | 0.03436±0.01201 | 1.000 | 0.986 | 0.676 | 0.647 | 0.680 | 0.921 |
| PC 40:3 | 0.20368±0.01467 | 0.13336±0.07313 | 0.1829±0.01869 | 0.17192±0.01197 | 0.18603±0.01508 | 0.19628±0.02253 | 0.18585±0.01557 | 0.18391±0.01416 | 0.16121±0.04851 | 0.18863±0.0132 | 0.1908±0.0192 | 1.000 | 1.394 | 1.006 | 0.938 | 1.014 | 0.972 |
| PC 40:5 | 2.30588±0.22557 | 1.4963±0.30195 | 1.6551±0.18643 | 1.91352±0.30059 | 1.72353±0.18398 | 1.04799±0.07847 | 2.2074±0.22981 | 1.64109±0.2453 | 1.59327±0.24655 | 2.2164±0.27747 | 1.62979±0.21123 | 1.000 | 1.475 | 0.992 | 0.833 | 1.286 | 1.555 |
| PC 40:7 | 7.57099±0.98681 | 5.96593±0.73614 | 6.86707±0.67483 | 6.7757±0.43859 | 7.0183±1.08441 | 3.03077±0.51883 | 6.98919±1.22851 | 5.76831±0.43635 | 5.15085±0.95166 | 5.54555±0.88028 | 4.24017±0.97162 | 1.000 | 1.172 | 0.840 | 0.760 | 0.790 | 1.399 |
| PC 40:8 | 1.67333±0.19383 | 0.75839±0.1327 | 0.87479±0.07816 | 0.81744±0.07936 | 0.74452±0.09404 | 0.32697±0.05098 | 1.20393±0.32461 | 1.09232±0.19448 | 0.99996±0.22868 | 1.09549±0.15207 | 0.83288±0.25572 | 1.000 | 1.587 | 1.249 | 1.223 | 1.471 | 2.547 |
| PC 42:6 | 0.13169±0.03124 | 0.09976±0.01927 | 0.11488±0.01853 | 0.09123±0.01277 | 0.07149±0.01288 | 0.16438±0.03464 | 0.04948±0.01434 | 0.04298±0.00526 | 0.04682±0.01246 | 0.0513±0.01137 | 0.06524±0.01946 | 1.000 | 0.496 | 0.374 | 0.513 | 0.718 | 0.397 |
| PC 42:7 | 0.1563±0.01471 | 0.1559±0.01818 | 0.16478±0.02089 | 0.17498±0.01519 | 0.13874±0.02283 | 0.14148±0.03158 | 0.11353±0.02524 | 0.09787±0.00311 | 0.0955±0.01794 | 0.11301±0.01346 | 0.11429±0.0274 | 1.000 | 0.728 | 0.594 | 0.546 | 0.815 | 0.808 |
| PC 42:8 | 0.08809±0.02169 | 0.0562±0.00656 | 0.06141±0.00641 | 0.06266±0.0083 | 0.05869±0.00888 | 0.06354±0.00961 | 0.07367±0.01802 | 0.06909±0.00776 | 0.07705±0.01996 | 0.10182±0.01935 | 0.08721±0.01151 | 1.000 | 1.311 | 1.125 | 1.230 | 1.735 | 1.373 |
| LPC 14:0 | 0.00517±0.00109 | 0.00695±0.00132 | 0.0059±0.0012 | 0.00741±0.00088 | 0.00537±0.00114 | 0.00485±0.00051 | 0.00282±0.00048 | 0.00293±0.00067 | 0.00311±0.00057 | 0.00522±0.00158 | 0.0037±0.00154 | 1.000 | 0.406 | 0.498 | 0.419 | 0.972 | 0.762 |
| LPC 16:0 | 1.72962±0.24849 | 1.63359±0.22886 | 1.63735±0.16167 | 1.52251±0.27079 | 1.54936±0.17833 | 1.37484±0.13106 | 1.27854±0.21922 | 1.20053±0.2627 | 1.33095±0.25623 | 2.00128±0.27757 | 1.53249±0.42712 | 1.000 | 0.783 | 0.733 | 0.874 | 1.292 | 1.115 |
| LPC 16:1 | 0.05042±0.00968 | 0.07233±0.01116 | 0.06448±0.00852 | 0.06355±0.01546 | 0.05694±0.00949 | 0.04265±0.00688 | 0.01683±0.00335 | 0.01563±0.00411 | 0.02015±0.0038 | 0.02382±0.00761 | 0.01758±0.00865 | 1.000 | 0.233 | 0.242 | 0.317 | 0.418 | 0.412 |
| LPC 18:0 | 1.00099±0.17855 | 0.7515±0.06173 | 0.75557±0.02853 | 0.80456±0.13901 | 0.71986±0.05974 | 0.63544±0.05243 | 0.75839±0.08585 | 0.72516±0.06242 | 0.8286±0.09253 | 1.19018±0.14696 | 0.92223±0.16038 | 1.000 | 1.009 | 0.960 | 1.030 | 1.653 | 1.451 |
| LPC 18:1 | 0.33935±0.05423 | 0.39088±0.05773 | 0.3518±0.01027 | 0.367±0.04163 | 0.30695±0.02432 | 0.31194±0.04873 | 0.17014±0.01732 | 0.15587±0.03426 | 0.18602±0.05041 | 0.23709±0.03739 | 0.17903±0.0546 | 1.000 | 0.435 | 0.443 | 0.507 | 0.772 | 0.574 |
| LPC 18:2 | 1.07759±0.29681 | 1.35071±0.18187 | 1.41709±0.08723 | 1.34673±0.13465 | 1.19148±0.09269 | 1.00784±0.18843 | 0.69169±0.09129 | 0.57527±0.21263 | 0.62904±0.23204 | 0.80374±0.19458 | 0.54193±0.16402 | 1.000 | 0.512 | 0.406 | 0.467 | 0.675 | 0.538 |
| LPC 18:3 | 0.03329±0.01015 | 0.04232±0.00517 | 0.041±0.00472 | 0.03347±0.00622 | 0.02755±0.00225 | 0.02078±0.00347 | 0.01603±0.00197 | 0.0118±0.00459 | 0.01178±0.00465 | 0.01458±0.00563 | 0.00789±0.00257 | 1.000 | 0.379 | 0.288 | 0.352 | 0.529 | 0.380 |
| LPC 19:0 | 0.02256±0.00363 | 0.02738±0.00302 | 0.02759±0.00273 | 0.02844±0.00262 | 0.02322±0.00279 | 0.02044±0.00319 | 0.01972±0.00256 | 0.01899±0.00225 | 0.0186±0.00223 | 0.02318±0.00551 | 0.01814±0.00268 | 1.000 | 0.720 | 0.688 | 0.654 | 0.999 | 0.887 |
| LPC 20:0 | 0.02209±0.00624 | 0.01986±0.00147 | 0.02139±0.003 | 0.01772±0.00255 | 0.01295±0.00109 | 0.02318±0.00761 | 0.00935±0.00225 | 0.00965±0.00105 | 0.01084±0.00197 | 0.01203±0.00191 | 0.01363±0.00335 | 1.000 | 0.471 | 0.451 | 0.612 | 0.929 | 0.588 |
| LPC 20:1 | 0.00995±0.00148 | 0.01143±0.0023 | 0.01104±0.00148 | 0.01061±0.00128 | 0.00825±0.00192 | 0.00873±0.00238 | 0.0056±0.00114 | 0.00571±0.0016 | 0.00623±0.00093 | 0.00821±0.00153 | 0.00709±0.00197 | 1.000 | 0.490 | 0.517 | 0.587 | 0.995 | 0.812 |
| LPC 20:2 | 0.0094±0.0014 | 0.00693±0.00057 | 0.00738±0.00028 | 0.00721±0.00179 | 0.0063±0.00134 | 0.01135±0.0027 | 0.00466±0.00068 | 0.00383±0.00106 | 0.00536±0.00121 | 0.00851±0.00121 | 0.00643±0.00161 | 1.000 | 0.671 | 0.519 | 0.743 | 1.349 | 0.567 |
| LPC 20:3 | 0.08791±0.02209 | 0.05667±0.00635 | 0.05837±0.00391 | 0.05258±0.01138 | 0.04638±0.00896 | 0.09647±0.00589 | 0.03169±0.00633 | 0.02782±0.00863 | 0.03051±0.00948 | 0.03732±0.00537 | 0.03662±0.01397 | 1.000 | 0.559 | 0.477 | 0.580 | 0.805 | 0.380 |
| LPC 20:4 | 0.48184±0.09082 | 0.43017±0.03455 | 0.46567±0.03044 | 0.43647±0.10952 | 0.41395±0.07462 | 0.29395±0.03314 | 0.22031±0.04368 | 0.18494±0.07931 | 0.18467±0.07642 | 0.2825±0.0714 | 0.17966±0.06799 | 1.000 | 0.512 | 0.397 | 0.423 | 0.682 | 0.611 |
| LPC 22:6 | 0.63836±0.16969 | 0.58737±0.05966 | 0.64892±0.07074 | 0.57084±0.08096 | 0.56738±0.0778 | 0.48912±0.07278 | 0.3668±0.05748 | 0.32172±0.12252 | 0.29768±0.13378 | 0.47232±0.11112 | 0.2928±0.11734 | 1.000 | 0.624 | 0.496 | 0.521 | 0.832 | 0.599 |
| LPC 24:0 | 0.01027±0.00086 | 0.0104±0.0023 | 0.01098±0.00143 | 0.01098±0.00289 | 0.01065±0.00072 | 0.00715±0.00048 | 0.00856±0.0016 | 0.00808±0.00062 | 0.01024±0.00095 | 0.01426±0.00379 | 0.0124±0.00236 | 1.000 | 0.823 | 0.736 | 0.933 | 1.339 | 1.734 |
| PC O-34:1 | 0.19841±0.0408 | 0.15312±0.02318 | 0.18045±0.02915 | 0.18907±0.03165 | 0.16857±0.02492 | 0.12459±0.02961 | 0.1897±0.0243 | 0.18053±0.0289 | 0.20072±0.02817 | 0.25761±0.0256 | 0.34941±0.07767 | 1.000 | 1.239 | 1.000 | 1.062 | 1.528 | 2.805 |
| PC O-34:2 | 0.08092±0.02318 | 0.06429±0.01207 | 0.06545±0.01452 | 0.06612±0.00314 | 0.0542±0.00838 | 0.05022±0.02401 | 0.07545±0.01252 | 0.08637±0.01938 | 0.10185±0.01577 | 0.14791±0.02478 | 0.21219±0.07131 | 1.000 | 1.174 | 1.320 | 1.540 | 2.729 | 4.225 |
| PC O-36:3 | 0.03048±0.00967 | 0.02417±0.00489 | 0.02499±0.00695 | 0.02496±0.00217 | 0.02198±0.00396 | 0.02276±0.00646 | 0.03176±0.00384 | 0.03307±0.00615 | 0.03655±0.00632 | 0.05741±0.0067 | 0.07911±0.02534 | 1.000 | 1.314 | 1.324 | 1.464 | 2.612 | 3.476 |
| PC O-36:4 | 0.23065±0.05416 | 0.21118±0.04454 | 0.22271±0.03057 | 0.24827±0.02778 | 0.25912±0.06021 | 0.11463±0.0251 | 0.23059±0.03243 | 0.20883±0.02946 | 0.22153±0.02535 | 0.29066±0.03512 | 0.32282±0.03229 | 1.000 | 1.092 | 0.938 | 0.892 | 1.122 | 2.816 |
| PC O-38:5 | 0.26867±0.08124 | 0.26219±0.06347 | 0.27748±0.0425 | 0.30633±0.04412 | 0.32262±0.07977 | 0.14523±0.04113 | 0.27563±0.03585 | 0.2329±0.03562 | 0.23622±0.02784 | 0.30531±0.03133 | 0.28636±0.02309 | 1.000 | 1.051 | 0.839 | 0.771 | 0.946 | 1.972 |
| PC O-38:6 | 0.14394±0.02518 | 0.13725±0.02647 | 0.15652±0.02253 | 0.18843±0.0156 | 0.17746±0.02397 | 0.07813±0.01923 | 0.15276±0.03572 | 0.11895±0.01209 | 0.12455±0.01674 | 0.17016±0.03097 | 0.16803±0.01507 | 1.000 | 1.113 | 0.760 | 0.661 | 0.959 | 2.151 |
| LPC O-18:1 | 0.00447±0.00115 | 0.00382±0.00039 | 0.00349±0.00041 | 0.00397±0.00103 | 0.00395±0.00109 | 0.00276±0.00065 | 0.00521±0.00042 | 0.00465±0.00052 | 0.00493±0.00087 | 0.00574±0.00055 | 0.00635±0.00141 | 1.000 | 1.365 | 1.333 | 1.241 | 1.453 | 2.300 |
| LPC O-16:0 | 0.00689±0.00202 | 0.00545±0.00047 | 0.00557±0.00056 | 0.00583±0.00123 | 0.00541±0.00066 | 0.00475±0.00102 | 0.00731±0.00078 | 0.00767±0.00061 | 0.00871±0.00086 | 0.01228±0.00186 | 0.01381±0.00172 | 1.000 | 1.343 | 1.377 | 1.495 | 2.270 | 2.906 |
|  |  |  |  |  |  |  |  |  |  |  |  |  |  |  |  |  |  |
| PE 32:0 | 0.0406±0.0094 | 0.01623±0.00207 | 0.01903±0.00352 | 0.02139±0.00598 | 0.02573±0.006 | 0.03147±0.00446 | 0.01564±0.00365 | 0.01655±0.0033 | 0.02045±0.0084 | 0.03418±0.00866 | 0.05669±0.00972 | 1.000 | 0.964 | 0.870 | 0.956 | 1.328 | 1.802 |
| PE 32:1 | 0.16998±0.05977 | 0.12404±0.02422 | 0.10906±0.02443 | 0.12106±0.02362 | 0.13804±0.03582 | 0.11908±0.02992 | 0.04352±0.0105 | 0.04322±0.01196 | 0.03884±0.0118 | 0.03905±0.00663 | 0.06233±0.02524 | 1.000 | 0.351 | 0.396 | 0.321 | 0.283 | 0.523 |
| PE 34:1 | 1.57551±0.23152 | 1.27845±0.26887 | 1.14517±0.30467 | 1.35425±0.26617 | 1.46001±0.31148 | 1.23839±0.08323 | 0.62505±0.17414 | 0.52562±0.08579 | 0.69322±0.22819 | 0.86645±0.1875 | 1.36948±0.17518 | 1.000 | 0.489 | 0.459 | 0.512 | 0.593 | 1.106 |
| PE 34:2 | 12.8828±1.4739 | 11.65221±1.75894 | 13.1886±2.09294 | 11.76374±1.03886 | 12.61247±1.90738 | 7.19603±0.69897 | 9.88129±2.66094 | 9.35731±2.3157 | 9.83547±1.07976 | 9.37067±2.27914 | 11.01047±1.71053 | 1.000 | 0.848 | 0.709 | 0.837 | 0.743 | 1.530 |
| PE 34:3 | 0.27666±0.07596 | 0.30448±0.03983 | 0.29398±0.05175 | 0.29722±0.04319 | 0.27224±0.0508 | 0.11615±0.02158 | 0.12828±0.02414 | 0.12463±0.04067 | 0.1493±0.02651 | 0.06893±0.01582 | 0.07443±0.02487 | 1.000 | 0.421 | 0.424 | 0.502 | 0.253 | 0.641 |
| PE 34:4 | 0.0082±0.00263 | 0.00804±0.00079 | 0.00725±0.00216 | 0.00842±0.00107 | 0.00637±0.0019 | 0.00453±0.00483 | 0.0035±0.00117 | 0.00287±0.00078 | 0.00314±0.00111 | 0.00201±0.00063 | 0.00316±0.00259 | 1.000 | 0.436 | 0.396 | 0.373 | 0.315 | 0.697 |
| PE 36:1 | 0.49128±0.13389 | 0.50646±0.08297 | 0.43796±0.08983 | 0.76688±0.16399 | 0.49396±0.02775 | 0.5226±0.09141 | 0.35854±0.04208 | 0.31715±0.02553 | 0.35434±0.06052 | 0.50825±0.0236 | 0.55196±0.04596 | 1.000 | 0.708 | 0.724 | 0.462 | 1.029 | 1.056 |
| PE 36:2 | 9.33572±0.73202 | 6.35217±0.64019 | 7.13618±1.25857 | 7.78894±1.493 | 6.98368±0.99924 | 6.68207±1.08128 | 8.4026±1.34327 | 7.90285±1.91062 | 8.88389±0.89843 | 8.92061±1.70681 | 10.27224±0.9533 | 1.000 | 1.323 | 1.107 | 1.141 | 1.277 | 1.537 |
| PE 36:3 | 4.52637±0.66882 | 4.1376±0.43636 | 4.3573±1.13261 | 4.95054±0.82022 | 4.43421±0.77282 | 2.33835±0.64051 | 3.44156±0.62027 | 3.29346±1.25104 | 3.69792±0.7141 | 2.71196±0.42047 | 3.11434±0.40408 | 1.000 | 0.832 | 0.756 | 0.747 | 0.612 | 1.332 |
| PE 36:4 | 21.13367±3.76157 | 9.76124±1.61735 | 11.84433±1.3462 | 10.53413±1.36801 | 13.72464±2.23012 | 14.31383±4.13788 | 11.43993±3.65545 | 11.13947±2.81186 | 11.383±2.51536 | 12.34532±3.88062 | 16.79481±3.27764 | 1.000 | 1.172 | 0.940 | 1.081 | 0.900 | 1.173 |
| PE 36:5 | 0.94028±0.0961 | 0.71234±0.1173 | 0.83725±0.13872 | 0.76427±0.07247 | 0.85375±0.11032 | 0.66277±0.06996 | 0.73424±0.24392 | 0.72516±0.19961 | 0.80365±0.12732 | 0.73891±0.21412 | 0.9348±0.11226 | 1.000 | 1.031 | 0.866 | 1.052 | 0.865 | 1.410 |
| PE 36:6 | 0.04361±0.01252 | 0.04895±0.00637 | 0.04959±0.0098 | 0.04865±0.00448 | 0.04733±0.00506 | 0.02007±0.00107 | 0.02435±0.00434 | 0.02084±0.00878 | 0.02275±0.00659 | 0.01285±0.00431 | 0.01288±0.00368 | 1.000 | 0.497 | 0.420 | 0.468 | 0.272 | 0.642 |
| PE 38:3 | 0.74248±0.1149 | 0.17634±0.02921 | 0.18513±0.01655 | 0.16855±0.02567 | 0.16854±0.05025 | 0.50046±0.0852 | 0.31297±0.06889 | 0.28608±0.05029 | 0.34121±0.06609 | 0.40291±0.12853 | 0.7024±0.21898 | 1.000 | 1.775 | 1.545 | 2.024 | 2.391 | 1.403 |
| PE 38:4 | 62.58679±11.24637 | 28.12836±6.15468 | 30.29166±5.62268 | 32.3699±6.80164 | 36.61622±5.53416 | 40.91952±9.18003 | 45.91448±14.74562 | 45.54011±13.05367 | 44.99623±10.48609 | 56.26754±20.46132 | 72.59497±12.94463 | 1.000 | 1.632 | 1.503 | 1.390 | 1.537 | 1.774 |
| PE 38:5 | 12.29722±4.39338 | 5.4648±0.93098 | 6.50216±0.87327 | 5.56074±0.68951 | 6.91006±1.70141 | 5.49108±1.42621 | 6.42093±2.01907 | 6.12031±1.79282 | 6.13669±1.55422 | 5.94532±1.66279 | 7.59712±1.74219 | 1.000 | 1.175 | 0.941 | 1.104 | 0.860 | 1.384 |
| PE 38:6 | 53.88443±7.33231 | 23.61105±2.69063 | 28.53572±4.45296 | 26.71547±3.62161 | 35.71062±7.92624 | 44.28921±0.9823 | 37.22643±11.49351 | 37.41662±6.93016 | 38.3874±7.92835 | 48.3996±14.94876 | 64.14774±12.76011 | 1.000 | 1.577 | 1.311 | 1.437 | 1.355 | 1.448 |
| PE 38:7 | 0.74947±0.22856 | 0.44809±0.06771 | 0.47638±0.06498 | 0.41898±0.08344 | 0.53391±0.11109 | 0.2429±0.02 | 0.33157±0.09298 | 0.30047±0.09176 | 0.27028±0.06478 | 0.2045±0.03924 | 0.22865±0.09075 | 1.000 | 0.740 | 0.631 | 0.645 | 0.383 | 0.941 |
| PE 38:8 | 0.06989±0.0207 | 0.03066±0.00585 | 0.04377±0.00739 | 0.03656±0.00417 | 0.04765±0.00575 | 0.02259±0.00528 | 0.03369±0.01081 | 0.03209±0.00612 | 0.04088±0.00944 | 0.04483±0.0099 | 0.0513±0.01507 | 1.000 | 1.099 | 0.733 | 1.118 | 0.941 | 2.270 |
| PE 40:3 | 0.05889±0.01907 | 0.04208±0.00686 | 0.05532±0.00916 | 0.04153±0.01113 | 0.04285±0.01393 | 0.05923±0.02975 | 0.01903±0.01312 | 0.01885±0.00921 | 0.0243±0.00797 | 0.02843±0.00642 | 0.02296±0.01915 | 1.000 | 0.452 | 0.341 | 0.585 | 0.663 | 0.388 |
| PE 40:4 | 0.20267±0.05091 | 0.0623±0.01261 | 0.06978±0.01285 | 0.07455±0.02387 | 0.08359±0.02407 | 0.14307±0.07459 | 0.17361±0.02583 | 0.12062±0.02539 | 0.12355±0.0185 | 0.21853±0.07614 | 0.30109±0.06467 | 1.000 | 2.787 | 1.729 | 1.657 | 2.614 | 2.104 |
| PE 40:5 | 1.1663±0.16559 | 0.61873±0.11638 | 0.72058±0.11345 | 0.69914±0.08768 | 0.81876±0.22267 | 0.76348±0.06461 | 0.74744±0.20618 | 0.69261±0.13055 | 0.71944±0.12941 | 0.92174±0.26196 | 1.10198±0.20618 | 1.000 | 1.208 | 0.961 | 1.029 | 1.126 | 1.443 |
| PE 40:6 | 20.55607±2.9405 | 8.58434±1.45161 | 10.02366±1.5456 | 10.25928±1.67618 | 12.92053±3.41538 | 12.01706±1.2482 | 18.02328±5.6994 | 15.78437±4.21461 | 16.59397±3.13418 | 23.90238±8.06845 | 26.69607±4.39433 | 1.000 | 2.100 | 1.575 | 1.617 | 1.850 | 2.222 |
| PE 40:7 | 9.29178±1.16909 | 3.71755±0.50502 | 4.53871±0.81743 | 4.31146±0.49771 | 5.51827±1.30302 | 4.02111±0.80523 | 6.7144±2.30619 | 6.38362±1.66581 | 5.81064±1.48778 | 7.01708±2.22418 | 7.52811±1.70948 | 1.000 | 1.806 | 1.406 | 1.348 | 1.272 | 1.872 |
| PE 40:8 | 0.7126±0.08053 | 0.1867±0.02916 | 0.25425±0.04798 | 0.22401±0.02941 | 0.25153±0.06863 | 0.12208±0.01356 | 0.49134±0.20148 | 0.47761±0.15797 | 0.43805±0.12114 | 0.4716±0.16679 | 0.48199±0.10694 | 1.000 | 2.632 | 1.878 | 1.956 | 1.875 | 3.948 |
| PE 42:8 | 0.1406±0.03301 | 0.04221±0.00813 | 0.05896±0.00688 | 0.05278±0.007 | 0.07006±0.02823 | 0.09602±0.0086 | 0.07906±0.03072 | 0.08154±0.01422 | 0.08905±0.02577 | 0.12466±0.0536 | 0.13568±0.0301 | 1.000 | 1.873 | 1.383 | 1.687 | 1.779 | 1.413 |
| LPE 16:0 | 0.49627±0.08524 | 0.61794±0.12582 | 0.59096±0.09658 | 0.52807±0.07798 | 0.51966±0.0673 | 0.37001±0.04748 | 0.37645±0.05902 | 0.43581±0.11734 | 0.5081±0.17476 | 0.4758±0.09047 | 0.32935±0.06195 | 1.000 | 0.609 | 0.737 | 0.962 | 0.916 | 0.890 |
| LPE 16:1 | 0.01712±0.00466 | 0.02597±0.00346 | 0.02463±0.00347 | 0.02095±0.00639 | 0.02116±0.00755 | 0.00752±0.00165 | 0.00826±0.00285 | 0.00802±0.00321 | 0.00882±0.00286 | 0.0083±0.00319 | 0.00345±0.001 | 1.000 | 0.318 | 0.326 | 0.421 | 0.392 | 0.459 |
| LPE 18:0 | 0.93733±0.17199 | 0.80054±0.04332 | 0.87544±0.08637 | 0.79417±0.08228 | 0.71707±0.06519 | 0.52045±0.01595 | 0.74094±0.17681 | 0.7394±0.21465 | 0.87169±0.23985 | 0.96297±0.16853 | 0.65155±0.09428 | 1.000 | 0.926 | 0.845 | 1.098 | 1.343 | 1.252 |
| LPE 18:1 | 0.24841±0.04783 | 0.28987±0.0238 | 0.28886±0.0379 | 0.24641±0.03481 | 0.22532±0.0159 | 0.12147±0.01733 | 0.16157±0.04212 | 0.15819±0.04048 | 0.18401±0.05622 | 0.18463±0.05066 | 0.1068±0.02558 | 1.000 | 0.557 | 0.548 | 0.747 | 0.819 | 0.879 |
| LPE 18:2 | 0.17338±0.04778 | 0.26403±0.03665 | 0.25896±0.02324 | 0.25644±0.03097 | 0.19983±0.0104 | 0.1186±0.01761 | 0.14208±0.02861 | 0.12119±0.0331 | 0.13805±0.0418 | 0.12595±0.03794 | 0.07415±0.01002 | 1.000 | 0.538 | 0.468 | 0.538 | 0.630 | 0.625 |
| LPE 18:3 | 0.00853±0.00199 | 0.0126±0.00258 | 0.01127±0.00154 | 0.00953±0.0016 | 0.00732±0.00068 | 0.00474±0.00122 | 0.00483±0.00098 | 0.00476±0.001 | 0.00434±0.00102 | 0.00411±0.00138 | 0.00192±0.00051 | 1.000 | 0.383 | 0.422 | 0.456 | 0.561 | 0.405 |
| LPE 20:4 | 0.57993±0.0866 | 0.52717±0.072 | 0.55666±0.04772 | 0.51978±0.04828 | 0.49195±0.08169 | 0.36739±0.01796 | 0.36137±0.06183 | 0.30238±0.08818 | 0.33856±0.07324 | 0.38011±0.08361 | 0.25068±0.05128 | 1.000 | 0.685 | 0.543 | 0.651 | 0.773 | 0.682 |
| LPE 22:6 | 0.63812±0.21724 | 0.70108±0.06139 | 0.77862±0.0475 | 0.66294±0.03525 | 0.70983±0.1195 | 0.47505±0.07215 | 0.54812±0.09238 | 0.4632±0.13936 | 0.42947±0.14586 | 0.54131±0.1382 | 0.28707±0.05825 | 1.000 | 0.782 | 0.595 | 0.648 | 0.763 | 0.604 |
| PE O-34:2 | 0.04691±0.00806 | 0.02682±0.00535 | 0.03169±0.00738 | 0.03531±0.00346 | 0.03007±0.00479 | 0.03129±0.00543 | 0.04653±0.00854 | 0.04166±0.01022 | 0.03484±0.00677 | 0.06258±0.02437 | 0.07361±0.02054 | 1.000 | 1.735 | 1.315 | 0.987 | 2.081 | 2.352 |
| PE O-36:2 | 0.0411±0.0086 | 0.02795±0.00208 | 0.02614±0.00519 | 0.03151±0.00474 | 0.02187±0.00331 | 0.01766±0.00623 | 0.03933±0.00933 | 0.04772±0.02504 | 0.03391±0.00622 | 0.03638±0.01181 | 0.06116±0.03395 | 1.000 | 1.407 | 1.826 | 1.076 | 1.664 | 3.462 |
| PE O-36:4 | 0.14441±0.03405 | 0.05916±0.01023 | 0.07292±0.01033 | 0.07719±0.01445 | 0.08968±0.02366 | 0.06481±0.02185 | 0.12077±0.01894 | 0.11001±0.0262 | 0.09775±0.02655 | 0.14314±0.03028 | 0.18492±0.03868 | 1.000 | 2.041 | 1.509 | 1.266 | 1.596 | 2.853 |
| PE O-36:5 | 1.39688±0.26463 | 0.64066±0.11402 | 0.74628±0.11554 | 0.79032±0.1595 | 0.9198±0.21067 | 0.68614±0.19561 | 1.14421±0.16787 | 1.10177±0.23487 | 1.0216±0.18216 | 1.45742±0.39689 | 1.85375±0.3828 | 1.000 | 1.786 | 1.476 | 1.293 | 1.584 | 2.702 |
| PE O-38:4 | 0.02887±0.009 | 0.02843±0.00561 | 0.02905±0.00567 | 0.03535±0.00616 | 0.02831±0.00382 | 0.02276±0.00709 | 0.02998±0.00381 | 0.02394±0.0039 | 0.02521±0.00311 | 0.02781±0.00239 | 0.02811±0.00552 | 1.000 | 1.054 | 0.824 | 0.713 | 0.982 | 1.235 |
| PE O-38:5 | 0.53935±0.09208 | 0.23942±0.04553 | 0.27969±0.04684 | 0.29275±0.07526 | 0.33668±0.0811 | 0.29622±0.08362 | 0.42073±0.07989 | 0.41666±0.07645 | 0.43152±0.09425 | 0.57997±0.17037 | 0.82106±0.15395 | 1.000 | 1.757 | 1.490 | 1.474 | 1.723 | 2.772 |
| PE O-38:6 | 0.54115±0.07645 | 0.26393±0.0548 | 0.30592±0.04319 | 0.32314±0.06766 | 0.37818±0.09572 | 0.25814±0.06517 | 0.46699±0.07105 | 0.4487±0.10119 | 0.39824±0.07558 | 0.59172±0.1653 | 0.73184±0.15517 | 1.000 | 1.769 | 1.467 | 1.232 | 1.565 | 2.835 |
| PE O-38:7 | 0.97052±0.08994 | 0.46143±0.06952 | 0.57903±0.11565 | 0.59225±0.11508 | 0.67648±0.16734 | 0.4178±0.06867 | 0.74664±0.15526 | 0.78188±0.23694 | 0.7073±0.1545 | 1.02049±0.37111 | 1.21012±0.20647 | 1.000 | 1.618 | 1.350 | 1.194 | 1.509 | 2.896 |
| PE O-40:6 | 0.05768±0.00755 | 0.03295±0.00788 | 0.03348±0.00352 | 0.04478±0.00883 | 0.03893±0.00632 | 0.04028±0.00665 | 0.05504±0.01156 | 0.06125±0.01824 | 0.05146±0.00913 | 0.07341±0.01237 | 0.09433±0.02401 | 1.000 | 1.671 | 1.829 | 1.149 | 1.886 | 2.342 |
| PE O-40:7 | 0.62548±0.08533 | 0.29661±0.04248 | 0.33173±0.056 | 0.34893±0.08933 | 0.37695±0.09136 | 0.30683±0.04998 | 0.46651±0.06981 | 0.48384±0.09719 | 0.44393±0.10356 | 0.61969±0.18151 | 0.76542±0.11287 | 1.000 | 1.573 | 1.459 | 1.272 | 1.644 | 2.495 |
